# Consensus on core domains for hand eczema trials: Signs, symptoms, control and quality of life

**DOI:** 10.1111/jdv.20671

**Published:** 2025-04-25

**Authors:** Henriette Rönsch, Karl Philipp Drewitz, Amber Reck Atwater, Detlef Becker, Philipp Bentz, Richard Brans, Tricia Chong, Heinrich Dickel, Peter Elsner, Ana M. Giménez‐Arnau, Fabrizio Guarneri, María Graciela Guzmán Perera, Sarah Ibrahim, Dimitra Koumaki, Jamie Koelbel, Francesca Larese Filon, Suzana Ljubojević Hadžavdić, Laura Loman, Mihaly Matura, Sonja Molin, Robert Ofenloch, Katharina Piontek, Radoslaw Spiewak, Anne Strunk, Margo Reeder, David Reissig, Thomas Rustemeyer, Marie‐Louise Schuttelaar, Dagmar Simon, Manon Sloot, Markus F. C. Steiner, Saïda Tongalaza, Skaidra Valiukevičienė, Maurice Waitek, Elke Weisshaar, Stefan Wöhrl, Doreen Wolff, Andrea Bauer, Christian Apfelbacher

**Affiliations:** ^1^ Department of Dermatology University Hospital Carl Gustav Carus, Technische Universität Dresden Dresden Germany; ^2^ Institute of Social Medicine and Health Systems Research Medical Faculty, Otto von Guericke University Magdeburg Germany; ^3^ Department of Dermatology George Washington University Washington DC USA; ^4^ Distinctive Dermatology Vienna Virginia USA; ^5^ Department of Dermatology University of Mainz Mainz Germany; ^6^ Division of Occupational Dermatology, Department of Dermatology Ruprecht‐Karls‐University Heidelberg Germany; ^7^ Department of Dermatology, Environmental Medicine and Health Theory Osnabrück University Osnabrück Germany; ^8^ Institute for Interdisciplinary Dermatologic Prevention and Rehabilitation (iDerm), Osnabrück University Osnabrück Germany; ^9^ National Skin Centre Singapore Singapore; ^10^ Department of Dermatology, Venereology and Allergology St. Josef Hospital, University Medical Center, Ruhr University Bochum Bochum Germany; ^11^ SRH Hospital Gera Gera Germany; ^12^ Department of Dermatology Hospital del Mar Research Institute, Universitat Pompeu Fabra Barcelona Spain; ^13^ Dermatology, Department of Clinical and Experimental Medicine University of Messina Messina Italy; ^14^ Dermatology Department Hospital Angeles del Pedregal Mexico City Mexico; ^15^ Department of Dermatology University of Wisconsin School of Medicine and Public Health Madison Wisconsin USA; ^16^ Dermatology Department University Hospital of Heraklion Heraklion Crete Greece; ^17^ Clinical Unit of Occupational Medicine University of Trieste Trieste Italy; ^18^ Department of Dermatology and Venereology, University Hospital Center Zagreb School of Medicine University of Zagreb Zagreb Croatia; ^19^ Department of Dermatology University Medical Center Groningen, University of Groningen Groningen The Netherlands; ^20^ Departments of Dermatology Norrlands University Hospital Umeå Sweden; ^21^ Örebro University Hospital Örebro Sweden; ^22^ Division of Dermatology Queen’s University Kingston Ontario Canada; ^23^ Department of Dermatology, Venerology and Allergy Charité‐Universitätsmedizin Berlin Berlin Germany; ^24^ Department of Experimental Dermatology and Cosmetology Jagiellonian University Medical College Krakow Poland; ^25^ Institute and Policlinic of Occupational and Social Medicine Faculty of Medicine, Technische Universität Dresden Dresden Germany; ^26^ Department of Dermatology, Allergology and Occupational Dermatology Amsterdam University Medical Center Amsterdam The Netherlands; ^27^ Department of Dermatology Inselspital, Bern University Hospital, University of Bern Bern Switzerland; ^28^ NHS Grampian Occupational Health Service Aberdeen UK; ^29^ Department of Skin and Venereal Diseases Medical Academy, Lithuanian University of Health Sciences Kaunas Lithuania; ^30^ Floridsdorf Allergy Center (FAZ) Vienna Austria

## Abstract

**Background:**

Hand eczema (HE) is a common and complex skin disease. A uniform set of core outcomes and related measures for use in clinical trials is lacking, making it difficult to compare results across HE studies.

**Objective:**

To reach consensus on a set of core domains and subdomains that should be measured in future therapeutic HE trials.

**Methods:**

In 2024, we conducted a two‐round online Delphi (eDelphi) survey among international HE experts, including physicians, patients and their relatives, researchers and industry representatives. A domain/subdomain was included in the core set when ≥80% of participants rated is as ‘critically important’; 50% agreement or less resulted in its exclusion. Results from 50% to 80% were deemed controversial and subject for further discussion. During a hybrid consensus meeting, the stakeholders reviewed, completed and, if necessary, revised the preliminary eDelphi consensus.

**Results:**

In the first and second round of the eDelphi, 208 and 134 persons, respectively, participated. Forty participants from 18 countries attended the consensus meeting. Consensus was reached to include the core domains ‘signs of HE’ (with five core subdomains), ‘symptoms of HE’ (two subdomains), ‘HE‐related quality of life’ (four subdomains) and ‘HE control over time’ (four subdomains). The subdomains ‘desquamation/scaling’ and ‘emotional impact/mental health’ remained controversial. Consensus was reached that the domains ‘skin barrier function’ and ‘patient‐reported treatment experience’ and 28 subdomains should not be part of the core outcome set.

**Conclusions:**

To produce comparable and meaningful results, future trials evaluating the effectiveness of HE treatments should measure signs and symptoms of HE, HE‐related quality of life and HE control over time as core outcome domains. The next step of the HE core outcome set initiative (HECOS) is to identify appropriate measurement instruments.

## Graphical abstract

A core domain set for hand eczema was developed in an international consensus study with an online Delphi survey and a consensus meeting. Signs and symptoms of hand eczema, related quality of life and control over time should be measured in all future hand eczema therapeutic trials.

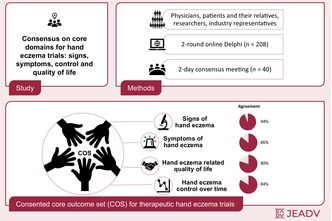

**Why was the study undertaken?**

- Therapeutic hand eczema (HE) trials require a consented core outcome set (COS) so that their results can be compared and synthesized.

**What does this study add?**

- International stakeholders have reached consensus about the core domains ‘signs of HE’ (with five core subdomains), ‘symptoms of HE’ (two subdomains), ‘HE‐related quality of life’ (four subdomains) and ‘HE control over time’ (four subdomains).

**What are the implications of this study for disease understanding and/or clinical care?**

- To enhance evidence‐based decision‐making, all future therapeutic HE trials should apply the consented COS.
- In future projects, suitable measurement instruments will be identified and added to the COS.

## INTRODUCTION

Hand eczema (HE) is a common skin disease that can seriously impair various aspects of life, including the ability to earn one's living.^1^, ^2^, ^3^ Therefore, there is a great need for effective HE treatments. Trials investigating the effectiveness of different HE treatments have applied diverging instruments to assess various outcomes.^4^ This impairs meaningful summaries and comparisons across trials, such as meta‐analyses, hampering evidence‐based decision‐making in clinical practice.^5^, ^6^

To address these concerns, the HE core outcome set (HECOS) initiative has initiated an international consensus process to agree on core domains and subdomains for a core outcome set (COS) to be applied in all future controlled trials (both randomized and non‐randomized, excluding laboratory experiments and observational studies) of therapeutic interventions that aim to ease the burden of HE (e.g. topical treatment, UV therapy, systemic treatment) including all types of HE in adults.^7^ In previous HECOS projects, 58 patients and 82 professional experts provided suggestions of potentially relevant domains.^8^

## MATERIALS AND METHODS

We conducted a two‐round online Delphi (eDelphi) survey in English, Dutch, German, Italian and Spanish (19 February 2024 to 25 June 2024) with Welphi (Decision Eyes, Lisbon, Portugal, 2024, www.welphi.com). This was followed by a hybrid (in presence and virtual) consensus meeting in Dresden, Germany, 3–4 September 2024. A protocol was published a priori.^7^

The institutional review board of the Technische Universität Dresden, Medical Faculty, reviewed the study protocol and study procedures and raised no objections to conduct the study (BO‐EK‐284062023, 18 August 2023). The study was registered in the Core Outcome Measures in Effectiveness Trials database (https://www.comet‐initiative.org/Studies/Details/1405). All participants provided their written informed consent to participate in the eDelphi or consensus meeting, respectively. We report our results according to the COS‐STAR (Core Outcome Set‐STAndards for Reporting) guideline and the checklist by Sinha et al.^9^, ^10^

### Participants

Potential experts with professional experience with HE were invited primarily by email or personally and were encouraged to forward our invitation (snowballing). A list of email addresses was compiled by the steering group based on previous collaborations, publications in the area of HE and websites of pharmaceutical or cosmetics companies. We thus reached out to physicians, researchers, and representatives of pharma or cosmetics companies. Patients were invited personally at the HECOS centres in Groningen (NL), Dresden, Heidelberg, Osnabrück (DE), Trieste (IT), Madison (Wisconsin, US) and Barcelona (ES). We offered participating patients or their relatives an incentive by covering their travel expenses for participating in the consensus meeting in person. All adults who had an interest in a COS for HE were eligible to participate.

### eDelphi

Based on previous projects,^8^ the steering group prepared an eDelphi questionnaire with 42 subdomains, grouped in seven overarching domains (Appendix S1). Participants of the eDelphi were asked to be ‘as selective as possible to keep the number of items in the core domain set as small as possible and represent only essential domains.’ In both rounds, we used a 6‐point scale: ‘How important is it to assess [domain or subdomain] to measure the effects of therapies in all future HE trials?’ (1–2: ‘not important’; 3–4: ‘important but not critical’; 5–6: ‘critically important, should be included in the core domain set’). During the first round, participants were permitted to suggest additional items; these were included in the second round after the steering committee confirmed that they were (A) not yet covered by an existing item, and (B) in an effectiveness domain. Items reaching criteria for consensus ‘in’ (included in the COS) or ‘out’ (excluded from the COS) during the first round (Table 1) were generally not presented again in the second round; the steering group decided to present subdomains again if they were particularly important for patients in the first round. Participants were informed about these consented items and received graphical information about all remaining items in the second round (Appendix S1, p. 17), including their own replies, encouraging them to re‐assess their rating in the spirit of consensus and paying particular attention to patients' ratings. Participants were not informed about the identity of other participants. Only participants of the first round were invited to the second, including those who did not rate all items.

**TABLE 1:** Consensus rules for eDelphi and consensus meeting.

Consensus classification	Description	Definition
Consensus ‘in’	Consensus that outcome should be included in the core outcome set	80% or more of participants rating the outcome critically important
Consensus ‘out’	Consensus that outcome should not be included in the core outcomes set	50% or fewer rating critically important
No consensus	Uncertainty about importance of outcome	50.1%–79.9% rating critically important

*Note*: Patient veto (only in consensus meeting): If a patient has a serious objection about any decision, the meeting will be paused for approx. 10 min. All patients get together and see if they unite behind this objection with ≥80%.

### Consensus meeting

All participants who completed both rounds were invited to the consensus meeting. The steering group decided to allow further stakeholders to join the meeting as well; they were invited as described above. We provided a document with the eDelphi results and a glossary before the meeting (Appendices S2 and S3).

**FIGURE 1:**
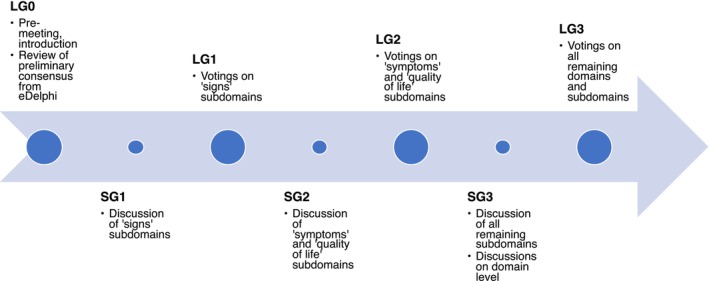
Course of the consensus meeting, LG: large group session; SG: small group discussion.

During a 1‐h premeeting followed by an introductory session, participants were provided with a summary of the preceding results of the eDelphi and upcoming tasks (Figure 1). The participants reviewed all items that had reached preliminary consensus during the eDelphi and were encouraged to propose that these be regarded as controversial items (large group 0, LG0) for further discussion. In the absence of a pre‐defined rule, an 80% threshold was ad hoc applied, which acknowledges the assessments of the many individuals who contributed to the eDelphi.

After this, controversial items of a certain domain were discussed primarily in small groups (SG1–SG3). The groups' speakers summarized the discussions in subsequent large group sessions (LG1–LG3), which was followed by voting about inclusion/exclusion of each controversial item (Table 1), or continued discussion as needed. We asked, ‘How important is it that [domain or subdomain] is included in the core domain set?’ (A: ‘not particularly important’, B: ‘important’, C: ‘critically important’). If consensus ‘in’ or ‘out’ was not achieved as defined in Table 1, voting was repeated once, which gave participants the opportunity to consider the group's ratings in their final vote. We utilized the platform eduVote (SimpleSoft, Braunschweig, Germany, www.eduvote.de); participants voted anonymously, using their own mobile devices.

## RESULTS

### eDelphi

**FIGURE 2:**
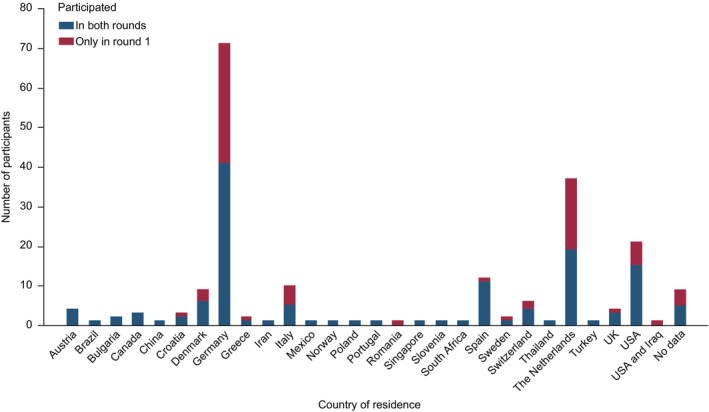
Country of residence of eDelphi participants.

**FIGURE 3:**
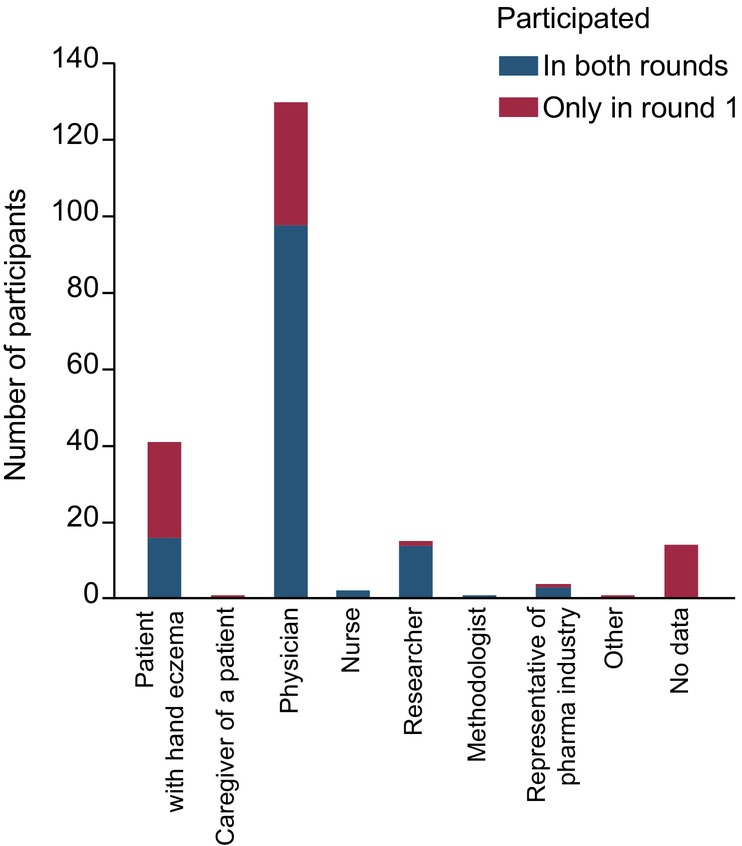
Experience with hand eczema of eDelphi participants.

In round 1, 208 stakeholders participated by rating at least one item. Their age ranged from 19 to 78 years (median: 53 years). Data on their experience with HE and country of residence are given in Figures 2 and 3. Out of the round 1 participants, 134 (64%) took part in round 2. Out of those who rated at least one item of the respective round, 28 participants (13%) did not complete the first round and eight (6%) did not complete the second round.

Among patients who were invited to participate in the eDelphi voting, reasons to decline included lack of interest, time expenditure, complexity of the study and technical issues. Patients were more likely to complete the first round of the eDelphi if a tablet or computer was made available for this purpose at the centres.

Participants reached consensus ‘in’ for the domains ‘signs of HE’, ‘symptoms of HE’, ‘HE‐related quality of life’, ‘HE control over time’ and 15 subdomains. They reached consensus ‘out’ for the domains ‘skin barrier function’, ‘patient‐reported treatment experience’ and 28 subdomains. Details are provided in Table 2. The distribution of eDelphi ratings is provided in Appendix S4. Appendix S5 compares round 1 results of patients who were lost/not lost to follow‐up in round 2.

**TABLE 2:** Percentage of ‘critically important’ ratings per (sub‐)domain during eDelphi and consensus meeting.

	Critically important for patients/caregivers		Critically important for all other participants^a^		Critically important for all participants (combined)^b^		
	Round 1	Round 2	Round 1	Round 2	Round 1	Round 2	Meeting
*n* (item dependent)	36–41	14–16	137–152	112–118	180–205	126–134	31–35
Domain ‘signs of HE’	88%	–	97%	–	94%	–	–
Domain ‘symptoms of HE’	93%	–	85%	–	86%	–	–
Domain ‘HE‐related quality of life’	76%	–	79.7%	–	80%	–	–
Domain ‘skin barrier function’	76%	–	32%	–	43%	–	–
Domain ‘patient‐reported treatment experience’	71%	50%	47%	59%	54%	58%	47%
Domain ‘HE control over time’	83%	–	83%	–	84%	–	–
Signs of HE							
Erythema (redness)	78%	67%	76%	82%	76%	80%	–
Infiltration (elevated skin)	77%	67%	81%	90%	79.8%	87%	–
Edema (swelling)	78%	79%	58%	61%	62%	63%	28%
Vesicles (blisters)	86%	–	85%	–	85%	–	–
Erosions, excoriation (scratch marks)	69%	64%	70%	77%	70%	76%	19%
Fissures, rhagades (cracks)	86%	–	88%	–	87%	–	–
Oozing (clear fluid that comes out of the skin)	89%	86%	53%	62%	61%	65%	9%
Bleeding/crusting	82%	79%	43%	49%	51%	52%	19%
Desquamation/scaling	73%	67%	62%	66%	65%	66%	74%
Lichenification (thickened hard skin)	66%	50%	72%	75%	70%	72%	25%
Hyperkeratosis (rough and scaly patches of skin)	76%	60%	58%	68%	62%	67%	84%
Dry skin	69%	53%	40%	40%	47%	42%	–
Nail changes	50%	–	38%	–	41%	–	–
Symptoms of HE							
Aching/pain	81%	80%	70%	77%	72%	78%	91%
Prickling	56%	–	21%	–	28%	–	–
Stinging	71%	–	30%	–	38%	–	–
Burning	86%	79%	39%	46%	50%	50%	14%
Pruritus (itching)	91%	–	93%	–	91%	–	–
Sensitive skin	77%	71%	29%	27%	39%	32%	–
Tight skin, impaired skin flexibility	69%	50%	32%	30%	40%	32%	–
HE‐related quality of life							
Physical hand functioning	84%	–	83%	–	83%	–	–
Ability to work or study	76%	–	88%	–	85%	–	–
Ability to take care of oneself or family	76%	–	82%	–	80%	–	–
Ability to practice leisure activities	57%	47%	64%	61%	63%	59%	43%
Extra efforts	68%	40%	34%	34%	42%	35%	–
Emotional impact	84%	67%	66%	76%	70%	75%	53%
Psychosocial impact	68%	67%	66%	72%	66%	71%	88%
Financial impact of HE	38%	–	39%	–	40%	–	–
Problem for loved ones	33%	–	23%	–	25%	–	–
Not having to think about hands, having the mind free for other things	54%	–	23%	–	30%	–	–
Conscious or unconscious, automatic scratching	67%	27%	30%	25%	38%	26%	–
Sleep disturbances	74%	46%	67%	75%	68%	72%	3%
Skin appearance, attractiveness of the skin	64%	40%	37%	30%	44%	31%	–
Skin barrier function							
Transepidermal water loss (TEWL)	69%	–	18%	–	27%	–	–
Patient‐reported treatment experience							
Treatment satisfaction	81%	71%	63%	78%	68%	77%	24%
Treatment tolerability	83%	93%	70%	83%	73%	84%	–
HE control over time							
Patient global assessment of treatment response	89%	87%	73%	85%	77%	85%	–
Period of time that is free of signs or symptoms of HE	69%	80%	67%	74%	67%	75%	34%
Number of flares in a given time	78%	60%	74%	83%	75%	80%	–
Area affected	74%	67%	72%	82%	72%	80%	–
Number of unscheduled doctor visits	–	33%	–	26%	–	27%	–
Use of additional medication or care products	–	86%	–	60%	–	63%	24%
Global assessment of treatment response by medical staff	–	80%	–	63%	–	65%	30%
Other							
Cure	86%	93%	78%	87%	79.9%	87%	13%
Chronicity	83%	93%	78%	90%	79%	90%	13%

*Note*: Green/red cell: Item was included in/excluded from core outcome set at this stage.

Abbreviation: HE, hand eczema.

^a^ Excluding stakeholders who did not provide this information.

^b^ Including stakeholders who did not provide this information. Therefore, the combined percentages can be larger or smaller than in either group.

Based on suggestions in round 1, the subdomains ‘number of unscheduled doctor visits’, ‘use of additional medication or care products’ and ‘global assessment of treatment response by medical staff’ were added to round 2 (see Appendix S6 for all suggestions). The HECOS coordinator (HR) and leads (CA, AB) reviewed all further comments; we summarized them for the consensus meeting (Appendix S7) and provided our responses between the eDelphi rounds (Appendix S8).

### Consensus meeting

Out of 45 stakeholders who registered, 35 attended both days of the consensus meeting in person or online, 5 participated on one day and 5 did not participate. The number of voters varied between 31 and 35 (some participants arrived late or only attended parts of the meeting). The stakeholders came from 18 countries (Table 3).

**TABLE 3:** Consensus meeting participants.

Country	*n*
Austria	1
Brazil	1
Canada	1
Denmark	1
Germany	15
Greece	1
Italy	3
Lithuania	2
Mexico	1
Poland	1
Romania	1
Singapore	1
Sweden	1
Switzerland	1
The Netherlands	4
Turkey	2
UK	2
USA	1
Experience with hand eczema (multiple replies possible)	
Industry representative	3
Patient or relative	6
Physician	26
Researcher	19

Among patients who were invited to the consensus meeting, reasons to decline were working times, a lack of interest, insufficient English skills, reservations due to the unfamiliar setting, time constraints or insufficient monetary compensation.

**FIGURE 4:**
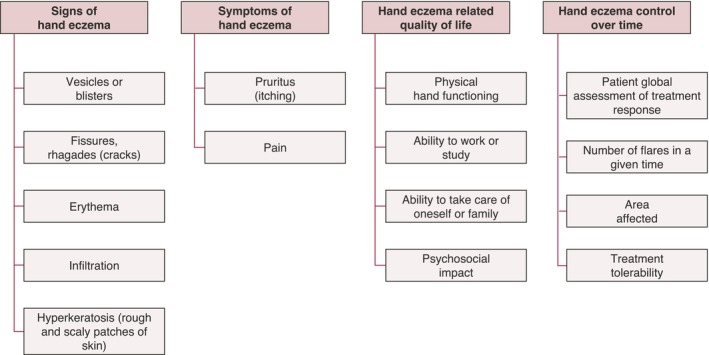
Core outcome domains and subdomains for therapeutic hand eczema trials.

The participants included four domains and 15 subdomains in the COS (Figure 4). Two domains (‘skin barrier function’, ‘patient‐reported treatment experience’) and 28 subdomains were excluded (Appendix S9). While reviewing all items with eDelphi consensus ‘in’ or ‘out’, six of them were suggested for re‐evaluation (see Appendix S10 for details and rationales).

### Domains

#### Signs of HE

The domain ‘signs of HE’ was included during the first round of the eDelphi with 94% agreement. Concerning ‘erythema’, two eDelphi participants mentioned that this is difficult to assess, particularly in patients with dark skin. The meeting participants widely agreed with the eDelphi decisions and took great care to add only few, essential core subdomains (Table 2). It was considered that ‘oozing’ and ‘bleeding/crusting’ were rated high by patients; however, the attending patient representatives did not share this view. It was argued that ‘oozing’ was covered by ‘vesicles’, and that bleeding (possibly resulting in crusting) was caused by either scratching or fissures and therefore an unspecific sign.

#### Symptoms of HE

The domain ‘symptoms of HE’ was included with 86% agreement in the first round of the eDelphi, along with the sub‐domain ‘pruritus’ (91%). The meeting participants added ‘aching/pain’ but renamed it to ‘pain’. After some discussion (Appendix S10), it was explicitly confirmed that ‘burning’ should not be part of the COS even though it was rated highly by patients. Fifty‐six meeting participants found ‘sleep disturbances’, ‘not particularly important’ and excluded it from the COS, reasoning that ‘pruritus’, which is the main reason for HE‐related sleep disturbances, was already a core subdomain and that other causes of sleep disturbances unrelated to HE might interfere.

#### HE‐related quality of life

The domain ‘HE‐related quality of life’ was rated as a core domain by 80% of participants in the first eDelphi round. Meeting participants noticed an overlap between the ‘emotional’ and ‘psychosocial impact of HE’ subdomains. While the latter was included as a core subdomain, the relevance of ‘emotional impact’ remained controversial. Several participants strongly underlined the importance of the ‘ability to practice leisure activities’; the majority found that this was important but not essential, arguing that it was controversial among patients who participated in the eDelphi.

#### Skin barrier function

Despite its high rating among patients, ‘skin barrier function’ was excluded during the eDelphi's first round (43% in favour of inclusion). The only subdomain was ‘transepidermal water loss’ (TEWL)^8^; only 27% of eDelphi participants found it essential, again with higher ratings among patients. Several participants commented that skin barrier function in general and TEWL in particular are difficult to assess because they require specific equipment and because such measurements vary across the hands' skin surface. Participants argued that these data are hard to interpret and not crucial for each therapeutic HE trial.

#### Patient‐reported treatment experience

The relevance of ‘patient‐reported treatment experience’ for the COS was controversial during the eDelphi, with 58% in favour during the second round. Even though the subdomain ‘treatment satisfaction’ almost reached the threshold for inclusion during the eDelphi, most meeting participants did not find it essential, arguing that it was already covered by other items, in particular ‘patient global assessment of treatment response’. Some participants nevertheless pointed out that ‘satisfaction’ refers to additional, important aspects. It was also argued that satisfaction is not an indicator of effectiveness. ‘Treatment tolerability’ was included during the eDelphi's second round (84% agreement), which was confirmed during the meeting after some discussion (Appendix S10). Meeting participants agreed to exclude the overarching domain ‘patient‐reported treatment experience’. As each sub‐domain required a domain, 88% agreed to move ‘treatment tolerability’ to the domain ‘HE control over time’, arguing that it could be seen as an aspect of control in therapeutic trials.

#### Hand eczema control over time

The domain ‘HE control over time’ was included during the eDelphi's first round (84% agreement) with comments underlining its importance in chronic HE. eDelphi participants included the subdomains ‘patient global assessment of treatment response’, ‘area affected’ and ‘number of flares in a given time’, which were rated similarly by patients and other stakeholders. Some participants pointed out that these subdomains needed refinement, such as defining whether percentage or hand regions (such as palms or fingertips) affected should be measured or how to define ‘flares’. During the meeting, we debated whether it would be possible to find a sufficiently clear definition for ‘flares’, so that patients would be able to count flares between visits. A patient underlined the importance of this item. Finally, ‘number of flares in a given time’ remained included. Meeting participants kept the decision to exclude the ‘number of unscheduled doctor visits’ because this outcome depended too much on external factors like the underlying healthcare system and accessibility. Moreover, they excluded three controversial ‘control’ subdomains, arguing that these were covered by included items such as signs and symptoms. Nevertheless, some participants remained convinced that these subdomains were critically important and just needed better definitions.

#### Uncategorized subdomains

An in‐depth discussion revealed that ‘chronicity’, which had reached consensus ‘in’ during the eDelphi (87% in round 2), was no longer considered an effectiveness outcome. Participants argued that, even though chronicity could be a highly relevant feature of HE, they would not expect it to change as result of a treatment. Similarly, ‘cure’ had been included in the eDelphi's second round (90%), but meeting participants decided to exclude ‘cure’. While some participants argued that there is no cure for (chronic) HE, others said that ‘cure’ meant absence of HE, which could be achieved by avoiding triggers in many types of HE. Finally, most participants agreed to exclude the item, either because cure was unachievable, or because ‘HE control’ already covered ‘freedom from HE’.

### Protocol deviations

Upon advice from the CHORD COUSIN Collaboration (C^3^), which is an international umbrella organization supporting COS initiatives in dermatology, the number of eDelphi rounds was reduced from three to two. The C^3^ experts reasoned that this would reduce the number of drop‐outs and would have little effect on the consensus itself, because it was unlikely that participants would change their mind in a third round if they had not already done so in the second. Moreover, it was decided against three specific statistical evaluations of the eDelphi results (mean changes in score, bimodality, intraclass correlation coefficient) because it was found that such results had no relevance for the COS.

## DISCUSSION

The HECOS initiative conducted an international consensus process which reached agreement that ‘signs of HE’, ‘symptoms of HE’, ‘HE‐related quality of life’ and ‘HE control over time’ should be measured as part of the core domain set for HE. These core domains are very similar to those developed for atopic dermatitis by the HOME (Harmonising Outcome Measures for Eczema) initiative.^11^ HECOS additionally specified 15 core subdomains, which will help to identify suitable measurement instruments in its subsequent phase.

Meeting participants agreed with most of the eDelphi decisions. The notable exceptions were ‘cure’ and ‘chronicity’. ‘Cure’ was excluded because other subdomains already cover freedom from HE sufficiently. ‘Chronicity’ was excluded because it cannot be an adequate measure of treatment effectiveness. We formally reached consensus to include ‘treatment tolerability’ and ‘flares’ based on the eDelphi, but the majority of meeting participants was not convinced that they are appropriate ‘control’ subdomains (Appendix S10). In general, there is some overlap between ‘control over time’ and other domains, which needs to be addressed in the future.

The next HECOS step will be a systematic review to identify and rate existing measurement instruments for the core domains, followed by another consensus process. Each core domain will be measured with one or more instruments, ideally addressing all core sub‐domains and no additional sub‐domains with a composite instrument. The still controversial subdomains ‘desquamation/scaling’ and ‘emotional impact’ are going to be addressed in this step as well.

### Strengths and limitations

By following established C^3^ methods,^12^ the HECOS consensus process has several strengths. First, we used a comprehensive list of candidate effectiveness outcomes items, which we previously identified by patient interviews and an expert survey.^8^ The steering group, encompassing experts on HE and COS development from five countries, carefully reviewed and categorized all suggested outcomes. Second, we offered the eDelphi in five languages and enabled online participation in the consensus meeting so that a wide range of stakeholders had the opportunity to participate.

Despite our efforts, patient participation was low throughout the consensus process. It was very difficult to convince them that their insights and assessments were essential. The majority were lost to follow‐up after the first round of the eDelphi process even though we highlighted that the second round would be decisive. Some items that were considered essential by most patients who dropped out were eventually excluded: ‘oozing’, ‘burning’, ‘TEWL’, ‘cure’ and ‘chronicity’. Our previous involvement of patients by means of personal interviews had been more successful.^8^ In such a setting, patients were willing to talk about essential outcomes. For future HECOS projects, we will consider face‐to‐face interviews rather than eDelphis and consensus meetings as the primary means of seeking patient input and feedback. Most participants of the eDelphi and consensus meeting were from Europe, with only five stakeholders (12.5%) from Asia and the Americas.

## CONCLUSIONS

As the result of an international eDelphi and consensus meeting, the HECOS initiative has developed a set of four core outcome domains with 15 subdomains. All future therapeutic HE trials should, as a minimum, measure (1) signs of HE (vesicles, fissures/rhagades, erythema, infiltration, hyperkeratosis), (2) symptoms of HE (pruritus, pain), (3) HE‐related quality of life (physical hand functioning, ability to work or study, ability to take care of oneself or family, psychosocial impact) and (4) HE control over time (patient global assessment of treatment response, number of flares in a given time, area affected, treatment tolerability). As a next step, HECOS will systematically identify or develop suitable measurement instruments for this core outcome set.

## AUTHOR CONTRIBUTIONS

Conceptualization, funding acquisition, project administration: Christian Apfelbacher, Andrea Bauer, Henriette Rönsch. Data curation, formal analysis, visualization, writing—original draft: Henriette Rönsch. Investigation: David Reissig, Henriette Rönsch and Saïda Tongalaza prepared and organized the eDelphi. Karl‐Philipp Drewitz and Henriette Rönsch prepared and organized the consensus meeting. Jamie Koelbel took the minutes of the consensus meeting. Validation: Christian Apfelbacher, Amber Reck Atwater, Andrea Bauer, Detlef Becker, Philipp Bentz, Richard Brans, Tricia Chong, Heinrich Dickel, Peter Elsner, Fabrizio Guarneri, María Graciela Guzmán Perera, Dimitra Koumaki, Francesca Larese Filon, Suzana Ljubojević Hadžavdić, Laura Loman, Mihaly Matura, Sonja Molin, Robert Ofenloch, Katharina Piontek, Radoslaw Spiewak, Anne Strunk, Thomas Rustemeyer, Henriette Rönsch, Dagmar Simon, Markus FC Steiner, Skaidra Valiukevičienė, Maurice Waitek, Elke Weisshaar, Stefan Wöhrl and Doreen Wolff participated in the consensus conference by discussing the eDelphi results and voting on the final core domains. Methodology: Christian Apfelbacher, Henriette Rönsch. Resources: Christian Apfelbacher, Andrea Bauer, Richard Brans, Karl‐Philipp Drewitz, Francesca Larese Filon, Ana M Gimenez‐Arnau, Sarah Ibrahim, Robert Ofenloch, Laura Loman, Margo Reeder, Henriette Rönsch, Thomas Rustemeyer, Marie‐Louise Schuttelaar, Dagmar Simon, Manon Sloot and Elke Weisshaar prepared the content of the eDelphi items and/or invited patients with hand eczema to participate in the eDelphi and consensus meeting. Software: David Reissig and Henriette Rönsch implemented the eDelphi questionnaire. Supervision: Christian Apfelbacher and Andrea Bauer. Writing—review and editing: All authors critically revised the manuscript and approved the final version to be published.

## FUNDING INFORMATION

European Academy of Dermatology and Venereology, Grant/Award Number: PPRC‐2023‐0024.

## CONFLICT OF INTEREST STATEMENT

Christian Apfelbacher was involved in the development and validation of the Quality Of Life in Hand Eczema Questionnaire (QOLHEQ). He has received honoraria for consultancy work from Dr. Wolff GmbH Bionorica, Sanofi, LEO Pharma, Incyte, Pfizer, Rheacell and IVDK and institutional funding from Dr. Wolff GmbH and Bionorica. Andrea Bauer is involved in clinical trials on hand eczema (LEO Pharma, Bristol Myers Squibb); she has been a speaker/advisor/investigator and/or received research funding from AbbVie, Almirall, Lilly, Galderma, Incyte, Janssen, LEO Pharma, L'Oréal, Pfizer, Regeneron and Sanofi. Richard Brans served as advisory board member and speaker for LEO Pharma. Detlef Becker received speaking honoraria from Sanofi‐Aventis. Heinrich Dickel has received honoraria as an advisory board member and/or speaker from Almirall Hermal GmbH, Stallergenes GmbH, LEO Pharma GmbH and Novartis Pharma GmbH. KP Drewitz is employed part‐time by Information Network of Departments of Dermatology, which is partly sponsored by Smart Practice Europe, the cosmetic industry or associations. Peter Elsner has been a speaker/advisor/investigator and/or received research funding from Almirall, Bayer, LEO Pharma, L'Oréal, Pierre Fabre, Sanofi and UCB. Ana M Gimenez‐Arnau is or recently was a speaker and/or advisor for and/or has received research funding from Almirall, Amgen, AstraZeneca, Avene, Blue‐ Print, Celldex, Escient Pharmaceutials, Genentech, GSK, Harmonic Bio, Instituto Carlos III‐ FEDER, Jaspers, Leo Pharma, Menarini, Mitsubishi Tanabe Pharma, Noucor, Novartis, Sanofi–Regeneron, Septerna, Servier, Thermo Fisher Scientific, Uriach Pharma. Fabrizio Guarneri has served as advisory board member for Leo Pharma. Francesca Larese Filon received a grant for 2 years' post‐doc from Unifarco–Santa Giustina–Belluno (Italy). Suzana Ljubojević Hadžavdić is PI for atopic dermatitis Clinical Study (Abbvie, Amgen, Nektar) and was PI for chronic spontaneous urticaria Clinical study (Novartis). She was lecturer for Novartis, Abbvie, Sanofi, Pliva, Bayer and Berlin‐Chemi. Sonja Molin has received honoraria as consultant/advisor or speaker and/or grants from Abbvie, Almirall, Aralez, Arcutis, Basilea, Bausch and Lomb, Bristol Myer Squibb, Boehringer‐Ingelheim, Evidera, Galderma, GSK, Incyte, Jamp Biopharma, LEO Pharma, Lilly, Novartis, Pfizer, Sanofi, Sun Pharma and UCB. Robert Ofenloch was involved in the development and validation of the QOLHEQ. Marie L.A. Schuttelaar was involved in the validation of the QOLHEQ, and is an advisor, consultant, speaker and/or investigator for Sanofi Genzyme, Regeneron Pharmaceuticals, Amgen, Incyte, Pfizer, Abbvie, LEO Pharma and Galderma. Dagmar Simon has been an investigator, advisory board member, or consultant for: AbbVie, Almirall, Amgen, AstraZeneca, Galderma, Incyte, LEO, Eli Lilly, Novartis, Pfizer and Sanofi Genzyme. Radoslaw Spiewak is a part‐time employee and shareholder at the Instytut Dermatologii, Krakow, Poland. He received lecture honoraria from the Polish Society of Allergology, the Polish Dermatological Society and Chiesi. His department received funds from Jagiellonian University Medical College, Krakow, Poland. Elke Weisshaar was involved in the development and validation of the QOLHEQ. The other authors have no conflicts of interest.

## ETHICAL APPROVAL

The institutional review board of the Technische Universität Dresden, Medical Faculty, Germany reviewed the study protocol and study procedures and did not raise objections to conduct the study (BO‐EK‐284062023, 2023‐08‐18).

## ETHICS STATEMENT

The participants acknowledged in this manuscript have given written informed consent to publication of their name and affiliation.

## Supporting information


Appendices S1‐S10

## Data Availability

The authors confirm that the data supporting the findings of this study are available within the article [and/or] its supplementary materials. Anonymized raw eDelphi data and meeting minutes are available from the corresponding author, HR, upon reasonable request.
