# Supplementary material for: Consensus on core domains for hand eczema trials: Signs, symptoms, control and quality of life
Source: J Eur Acad Dermatol Venereol. 2025 Apr 25;39(9):1588–99. doi: 10.1111/jdv.20671 (PMC12376261; doi:10.1111/jdv.20671)
Supplement: Supplementary file 1 — Appendices S1‐S10 [file JDV-39-1588-s001.zip › jdv20671-sup-0001-AppendixS1.pdf]

HECOS: Development of a hand eczema core outcome set

eDelphi survey for therapeutic domains and sub-domains

Round 1

Round 1: Introduction

Welcome to the HECOS survey - outcome domains in therapeutic hand eczema trials!

The purpose of this project is to develop a core domain set. This means an agreed minimum set of outcome domains that should be measured in trials on treatment of hand eczema.

We need your help to decide which outcomes are most important to be part of this core domain set. This survey addresses patients with hand eczema, their caregivers, and professional experts in the field of hand eczema. In this first round, we will start off with a few demographic questions about you and your background. Afterwards, **we will ask you to rate the importance of the presented domains regarding inclusion in the core domain set. Please be as selective as possible** in order to keep the number of items in the core domain set as small as possible and represent only the essential domains. This is important because it is burdensome for patients as well as researchers to assess a large set of core domains during a trial. **When providing your rating, please always consider WHICH of these domains are MOST IMPORTANT in trials of hand eczema treatment.**

In this first round, we ask your opinion concerning 6 broad hand eczema domains, followed by 44 specific sub-domains. The anticipated completion time is 30 minutes.

Please rate EACH item on a scale from 1 to 6:

- **1-2 represent an item that is NOT IMPORTANT**
- **3-4 represent an item which is IMPORTANT but NOT CRITICAL**
- **5-6 represent an item that is CRITICALLY IMPORTANT and SHOULD BE INCLUDED in the core domain set**
- **"Unable to score" in case you cannot provide a rating, for example if the item is not clear enough.**

In addition, there are free text boxes for every item. You may use them to justify your ratings, to tell us why you cannot rate an item, or to suggest other relevant outcomes. This provides valuable information to understand the reasoning behind a rating.
In the second round of the survey, the results of the whole group of participants from the previous round will be fed back to you. This offers you the chance to re-consider your personal rating. The goal of this procedure is that all participants finally reach an agreement about the **most important outcomes**. We apply the following consensus criteria:

- **Consensus 'in'**: rated 'critically important' by ≥ 80% of participants
- **Consensus 'out'**: rated 'critically important' by ≤ 50% of participants
- No consensus: all other cases (rated 'critically important by more than 50% but less than 80% of participants)

It is very likely that the questionnaire will be reduced considerably in round 2 because we will only include those items for which consensus 'in' or 'out' has not been reached already.

At the end of the questionnaire, you can suggest additional domains that have not been covered by the survey and you feel should be included in the next round. While the survey is still running, you can return to the survey at any time via the link sent to you and either continue to answer or make changes to your answers.

We would be grateful if you could rate all items and do not skip any of them. If you feel you are unable to score, please tick 'unable to score'.

This round will be online until ...

Thank you very much for your contribution!
On behalf of the HECOS initiative: Henriette Rönsch - Andrea Bauer - Christian Apfelbacher

Round 1: Questionnaire

Demographic data

Year of birth: ______
Country of residence: ______
Your experience with hand eczema:
(If several categories apply, please choose your most important background.)

☐ patient with hand eczema
☐ caregiver of a patient with hand eczema
☐ physician, specialty: ______
☐ researcher
☐ Methodologist
☐ journal editor
☐ nurse
☐ representative of pharma industry
☐ representative of regulatory authorities
☐ other

Domains

We will first ask you to rate six broad outcome domains. After this step, we ask for your ratings of the more detailed sub-domains.

**Domain 'signs of hand eczema'**<br>This means all the changes in the skin that a doctor, patient, or another person can look at during an examination.<br>*How important is it to assess **signs of hand eczema** to measure the effects of therapies in all future hand eczema trials?*						
not important		important but not critical		critically important, should be included in the core domain set		unable to score
1	2	3	4	5	6	
☐	☐	☐	☐	☐	☐	☐
Would you like to leave a comment or question? ______________________________________						

**Domain 'symptoms of hand eczema'**<br>This means all skin complaints that only the patient can feel and describe.<br>*How important is it to assess **symptoms of hand eczema** to measure the effects of therapies in all future hand eczema trials?*						
not important		important but not critical		critically important, should be included in the core domain set		unable to score
1	2	3	4	5	6	
☐	☐	☐	☐	☐	☐	☐
Would you like to leave a comment or question? ______________________________________						

**Domain 'hand eczema related quality of life'**

This means all aspects of life that are affected by hand eczema.<br>*How important is it to assess **hand eczema related quality of life** to measure the effects of therapies in all future hand eczema trials?*						
not important		important but not critical		critically important, should be included in the core domain set		unable to score
☐	☐	☐	☐	☐	☐	☐
1	2	3	4	5	6	
Would you like to leave a comment or question? ____						

**Domain 'skin barrier function'**<br>This is about whether that protective layer of the skin is working well or not.<br>*How important is it to assess **the skin barrier function** to measure the effects of therapies in all future hand eczema trials?*						
not important		important but not critical		critically important, should be included in the core domain set		unable to score
1	2	3	4	5	6	
☐	☐	☐	☐	☐	☐	☐
Would you like to leave a comment or question? ____						

**Domain 'patient-reported treatment experience'**<br>This is about how patients feel about the experience of their treatment overall.<br>*How important is it to assess **the patient-reported treatment experience** to measure the effects of therapies in all future hand eczema trials?*						
not important		important but not critical		critically important, should be included in the core domain set		unable to score
1	2	3	4	5	6	
☐	☐	☐	☐	☐	☐	☐
Would you like to leave a comment or question? ____						

**Domain 'hand eczema control over time'**<br>This is all about how well-controlled the hand eczema is over time. By regularly checking things like how severe it is or how often it flares up, we can figure out if hand eczema is being well controlled over time.<br>*How important is it to assess **hand eczema control over time** to measure the effects of therapies in all future hand eczema trials?*						
not important		important but not critical		critically important, should be included in the core domain set		unable to score
1	2	3	4	5	6	
☐	☐	☐	☐	☐	☐	☐
Would you like to leave a comment or question? ____						

Sub-domains within the domain 'signs of hand eczema'

**Erythema (redness)**

*How important is it to assess **erythema** to measure the effects of therapies in all future hand eczema trials?*						
not important		important but not critical		critically important, should be included in the core domain set		unable to score
1	2	3	4	5	6	
☐	☐	☐	☐	☐	☐	☐
Would you like to leave a comment or question? ____________________________						

**Infiltration (elevated skin): papules, patches, plaques, nodules**<br>This refers to the skin getting thicker, causing raised changes that you can feel, either with or without thick scaling. This thickening usually happens in specific areas, forming different types of bumps.<br>*How important is it to assess **infiltration** to measure the effects of therapies in all future hand eczema trials?*						
not important		important but not critical		critically important, should be included in the core domain set		unable to score
1	2	3	4	5	6	
☐	☐	☐	☐	☐	☐	☐
Would you like to leave a comment or question? ____________________________						

**Edema (swelling)**<br>This means that extra fluid builds up in the skin, making it puffy.<br>*How important is it to assess **edema** to measure the effects of therapies in all future hand eczema trials?*						
not important		important but not critical		critically important, should be included in the core domain set		unable to score
1	2	3	4	5	6	
☐	☐	☐	☐	☐	☐	☐
Would you like to leave a comment or question? ____________________________						

**Vesicles (blisters)**<br>*How important is it to assess **vesicles** to measure the effects of therapies in all future hand eczema trials?*						
not important		important but not critical		critically important, should be included in the core domain set		unable to score
1	2	3	4	5	6	
☐	☐	☐	☐	☐	☐	☐
Would you like to leave a comment or question? ____________________________						

**Erosions, excoriation (scratch marks)**<br>*How important is it to assess **erosions** to measure the effects of therapies in all future hand eczema trials?*						
not important		important but not critical		critically important, should be included in the core domain set		unable to score
1	2	3	4	5	6	

☐	☐	☐	☐	☐	☐	☐
Would you like to leave a comment or question? ____						

**Fissures, rhagades (cracks)**<br>*How important is it to assess **fissures** to measure the effects of therapies in all future hand eczema trials?*						
not important		important but not critical		critically important, should be included in the core domain set		unable to score
1	2	3	4	5	6	
☐	☐	☐	☐	☐	☐	☐
Would you like to leave a comment or question? ____						

**Oozing (clear fluid that comes out of the skin)**<br>*How important is it to assess **oozing** to measure the effects of therapies in all future hand eczema trials?*						
not important		important but not critical		critically important, should be included in the core domain set		unable to score
1	2	3	4	5	6	
☐	☐	☐	☐	☐	☐	☐
Would you like to leave a comment or question? ____						

**Bleeding/crusting**<br>*How important is it to assess **bleeding/crusting** to measure the effects of therapies in all future hand eczema trials?*						
not important		important but not critical		critically important, should be included in the core domain set		unable to score
1	2	3	4	5	6	
☐	☐	☐	☐	☐	☐	☐
Would you like to leave a comment or question? ____						

**Desquamation (when the outer layer of the skin peels off or flakes)**<br>*How important is it to assess **desquamation** to measure the effects of therapies in all future hand eczema trials?*						
not important		important but not critical		critically important, should be included in the core domain set		unable to score
1	2	3	4	5	6	
☐	☐	☐	☐	☐	☐	☐
Would you like to leave a comment or question? ____						

**Lichenification (thickened hard skin)**<br>**This means areas of the skin that are thick and hard from constantly scratching. This is different from “keratosis,” which are areas of the skin that are thick and scaly.**<br>*How important is it to assess **lichenification** to measure the effects of therapies in all future hand eczema trials?*

not important		important but not critical		critically important, should be included in the core domain set		unable to score
1	2	3	4	5	6	
☐	☐	☐	☐	☐	☐	☐
Would you like to leave a comment or question? ___						

**Keratosis (rough and scaly patches of skin)**<br>*How important is it to assess **keratosis** to measure the effects of therapies in all future hand eczema trials?*						
not important		important but not critical		critically important, should be included in the core domain set		unable to score
1	2	3	4	5	6	
☐	☐	☐	☐	☐	☐	☐
Would you like to leave a comment or question? ___						

**Dry skin**<br>*How important is it to assess **dry skin** to measure the effects of therapies in all future hand eczema trials?*						
not important		important but not critical		critically important, should be included in the core domain set		unable to score
1	2	3	4	5	6	
☐	☐	☐	☐	☐	☐	☐
Would you like to leave a comment or question? ___						

**Nail changes**<br>*How important is it to assess **nail changes** to measure the effects of therapies in all future hand eczema trials?*						
not important		important but not critical		critically important, should be included in the core domain set		unable to score
1	2	3	4	5	6	
☐	☐	☐	☐	☐	☐	☐
Would you like to leave a comment or question? ___						

Sub-domains within the domain “symptoms of hand eczema”

**Aching/pain**<br>*How important is it to assess **aching/pain** to measure the effects of therapies in all future hand eczema trials?*						
not important		important but not critical		critically important, should be included in the core domain set		unable to score
1	2	3	4	5	6	
☐	☐	☐	☐	☐	☐	☐
Would you like to leave a comment or question? ___						

Prickling

How important is it to assess ***prickling*** *to measure the effects of therapies in all future hand eczema trials?*

not important		important but not critical		critically important, should be included in the core domain set		unable to score
1	2	3	4	5	6	
☐	☐	☐	☐	☐	☐	☐
Would you like to leave a comment or question? ______________________________						

Stinging

How important is it to assess ***stinging*** *to measure the effects of therapies in all future hand eczema trials?*

not important		important but not critical		critically important, should be included in the core domain set		unable to score
1	2	3	4	5	6	
☐	☐	☐	☐	☐	☐	☐
Would you like to leave a comment or question? ______________________________						

Burning

How important is it to assess ***burning*** *to measure the effects of therapies in all future hand eczema trials?*

not important		important but not critical		critically important, should be included in the core domain set		unable to score
1	2	3	4	5	6	
☐	☐	☐	☐	☐	☐	☐
Would you like to leave a comment or question? ______________________________						

Pruritus (itching)

How important is it to assess ***pruritus*** *to measure the effects of therapies in all future hand eczema trials?*

not important		important but not critical		critically important, should be included in the core domain set		unable to score
1	2	3	4	5	6	
☐	☐	☐	☐	☐	☐	☐
Would you like to leave a comment or question? ______________________________						

Sensitive skin

This means when the skin feels uncomfortable—like stinging, burning, pain, itching, or tingling—when it is exposed to things like heat, cold, cosmetics, or water, even though these things usually should not make the skin feel that way.

How important is it to assess ***sensitive skin*** *to measure the effects of therapies in all future hand eczema trials?*

not important		important but not critical		critically important, should be included in the core domain set		unable to score
1	2	3	4	5	6	

☐	☐	☐	☐	☐	☐	☐
Would you like to leave a comment or question? ___						

**Tight skin, impaired skin flexibility**<br>*How important is it to assess **tight skin/impaired skin flexibility** to measure the effects of therapies in all future hand eczema trials?*						
not important		important but not critical		critically important, should be included in the core domain set		unable to score
1	2	3	4	5	6	
☐	☐	☐	☐	☐	☐	☐
Would you like to leave a comment or question? ___						

Sub-domains within the domain 'hand eczema related quality of life'

**Physical hand functioning (ability to perform various tasks and movements)**<br>This is about how well the hands can perform various tasks and movements. It includes actions like gripping, holding, and moving the fingers.<br>*How important is it to assess **physical hand functioning** to measure the effects of therapies in all future hand eczema trials?*						
not important		important but not critical		critically important, should be included in the core domain set		unable to score
1	2	3	4	5	6	
☐	☐	☐	☐	☐	☐	☐
Would you like to leave a comment or question? ___						

**Ability to work or study (e.g. sick leave, problems at work or study, discontinuation)**<br>*How important is it to assess **the ability to work** to measure the effects of therapies in all future hand eczema trials?*						
not important		important but not critical		critically important, should be included in the core domain set		unable to score
1	2	3	4	5	6	
☐	☐	☐	☐	☐	☐	☐
Would you like to leave a comment or question? ___						

**Ability to take care of oneself or family (housework, shopping, bathing, hygiene)**<br>*How important is it to assess **the ability to take care of oneself or family** to measure the effects of therapies in all future hand eczema trials?*						
not important		important but not critical		critically important, should be included in the core domain set		unable to score
1	2	3	4	5	6	
☐	☐	☐	☐	☐	☐	☐
Would you like to leave a comment or question? ___						

**Ability to practice leisure activities (e.g. hobbies, sport, gardening, social activities)**<br>*How important is it to assess **the ability to practice leisure activities** to measure the effects of therapies in all future hand eczema trials?*

not important		important but not critical		critically important, should be included in the core domain set		unable to score
1	2	3	4	5	6	
☐	☐	☐	☐	☐	☐	☐
Would you like to leave a comment or question? ____						

Extra efforts
This refers to any additional work patients have to take in order to care for the hand eczema. This includes having to attend doctor appointments, needing to use gloves, and having to spend time applying lotions and creams.
How important is it to assess extra efforts to measure the effects of therapies in all future hand eczema trials?

not important		important but not critical		critically important, should be included in the core domain set		unable to score
1	2	3	4	5	6	
☐	☐	☐	☐	☐	☐	☐
Would you like to leave a comment or question? ____						

Emotional impact (well-being, mood, unrest, depression, anger, annoyance, frustration, humiliation, shame, fears, worries, helplessness, acceptance, hope in treatment)
*How important is it to assess **the emotional impact** to measure the effects of therapies in all future hand eczema trials?*

not important		important but not critical		critically important, should be included in the core domain set		unable to score
1	2	3	4	5	6	
☐	☐	☐	☐	☐	☐	☐
Would you like to leave a comment or question? ____						

Psychosocial impact (e.g. concerning shaking hands, impairment of social contacts, relationships or sexual life, stigmatization, difficulties showing affection, self-consciousness, staying at home because of hand eczema, influence on clothing, withdrawal from social life)
*How important is it to assess **the psychological impact** to measure the effects of therapies in all future hand eczema trials?*

not important		important but not critical		critically important, should be included in the core domain set		unable to score
1	2	3	4	5	6	
☐	☐	☐	☐	☐	☐	☐
Would you like to leave a comment or question? ____						

Financial impact of hand eczema
This is about how the treatment a person gets can help them spend less money on things like over-the-counter products, extra treatments, doctor visits, travel, and not being able to work (which is lost income).
*How important is it to assess **the financial impact** to measure the effects of therapies in all future hand eczema trials?*

not important		important but not critical		critically important, should be included in the core domain set		unable to score
1	2	3	4	5	6	
☐	☐	☐	☐	☐	☐	☐
Would you like to leave a comment or question? ____						

**Problem for loved ones**<br>This refers to any impact that the hand eczema has on patients' loved ones.<br>*How important is it to assess **the problems for loved ones** to measure the effects of therapies in all future hand eczema trials?*						
not important		important but not critical		critically important, should be included in the core domain set		unable to score
1	2	3	4	5	6	
☐	☐	☐	☐	☐	☐	☐
Would you like to leave a comment or question? ____						

**Not having to think about hands, having the mind free for other things**<br>*How important is it to assess **not having to think about the hands/having the mind free for other things** to measure the effects of therapies in all future hand eczema trials?*						
not important		important but not critical		critically important, should be included in the core domain set		unable to score
1	2	3	4	5	6	
☐	☐	☐	☐	☐	☐	☐
Would you like to leave a comment or question? ____						

**Conscious or unconscious, automatic scratching**<br>*How important is it to assess **conscious or unconscious, automatic scratching** to measure the effects of therapies in all future hand eczema trials?*						
not important		important but not critical		critically important, should be included in the core domain set		unable to score
1	2	3	4	5	6	
☐	☐	☐	☐	☐	☐	☐
Would you like to leave a comment or question? ____						

**Sleep disturbances**<br>*How important is it to assess **sleep disturbances** to measure the effects of therapies in all future hand eczema trials?*						
not important		important but not critical		critically important, should be included in the core domain set		unable to score
1	2	3	4	5	6	
☐	☐	☐	☐	☐	☐	☐
Would you like to leave a comment or question? ____						

**Skin appearance, attractiveness of the skin, visibility of skin lesions**

*How important is it to assess **the skin appearance/attractiveness of the skin/visibility of skin lesions** to measure the effects of therapies in all future hand eczema trials?*						
not important		important but not critical		critically important, should be included in the core domain set		unable to score
1	2	3	4	5	6	
☐	☐	☐	☐	☐	☐	☐
Would you like to leave a comment or question? ____________________						

Sub-domains within the domains ‘skin barrier function’

**Transepidermal water loss (TEWL)**<br>Transepidermal water loss (TEWL) is when water is lost through the skin, like when it evaporates. If the skin is irritated due to hand eczema, it doesn't protect well, so more water is lost. Researchers measure TEWL with a special tool, and a high value might mean that the skin is damaged.<br>*How important is it to assess **transepidermal water loss** to measure the effects of therapies in all future hand eczema trials?*						
not important		important but not critical		critically important, should be included in the core domain set		unable to score
1	2	3	4	5	6	
☐	☐	☐	☐	☐	☐	☐
Would you like to leave a comment or question? ____________________						

Sub-domains within the domain ‘patient-reported treatment experience’

**Treatment satisfaction**<br>This means how satisfied someone is regarding the cosmetic and/or functional results of treatment.<br>*How important is it to assess **treatment satisfaction** to measure the effects of therapies in all future hand eczema trials?*						
not important		important but not critical		critically important, should be included in the core domain set		unable to score
1	2	3	4	5	6	
☐	☐	☐	☐	☐	☐	☐
Would you like to leave a comment or question? ____________________						

**Treatment tolerability**<br>Treatment tolerability means how well a person can handle and endure a particular treatment. It's about how comfortable or manageable the treatment is for the individual.<br>*How important is it to assess **treatment tolerability** to measure the effects of therapies in all future hand eczema trials?*						
not important		important but not critical		critically important, should be included in the core domain set		unable to score
1	2	3	4	5	6	
☐	☐	☐	☐	☐	☐	☐
Would you like to leave a comment or question? ____________________						

Sub-domains within the domain ‘hand eczema control over time’

Patient global assessment of treatment response
Global assessment of treatment response means looking at how well an entire treatment is working overall. It involves evaluating if the treatment is helping, making things better, or if there are still issues.
*How important is **a repeated patient global assessment of treatment response** to measure the effects of therapies in all future hand eczema trials?*

not important		important but not critical		critically important, should be included in the core domain set		unable to score
1	2	3	4	5	6	
☐	☐	☐	☐	☐	☐	☐

Would you like to leave a comment or question? ____

Period of time that is free of signs or symptoms of hand eczema
*How important is it to assess **the period of time that is free of signs or symptoms of hand eczema** to measure the effects of therapies in all future hand eczema trials?*

not important	important but not critical	critically important, should be included in the core domain set	unable to score			
1	2	3	4	5	6	
☐	☐	☐	☐	☐	☐	☐

Would you like to leave a comment or question? ____

Number of flares in a given time (e.g. number of flares in the past three months)
This means that it is recorded how often hand eczema gets worse during a given period of time.
*How important is it to assess **the number of flares repeatedly** to measure the effects of therapies in all future hand eczema trials?*

not important		important but not critical		critically important, should be included in the core domain set		unable to score
1	2	3	4	5	6	
☐	☐	☐	☐	☐	☐	☐

Would you like to leave a comment or question? ____

Area affected
This means how large of an area on the is affected with the condition. For example, it may only be one thumb, or the entire hand.
*How important is it to assess **the affected area** to measure the effects of therapies in all future hand eczema trials?*

not important		important but not critical		critically important, should be included in the core domain set		unable to score
1	2	3	4	5	6	
☐	☐	☐	☐	☐	☐	☐

Would you like to leave a comment or question? ____

Unclassified sub-domains

**Cure**<br>This means absence from signs and symptoms, normal looking skin of the hands.<br>*How important is it to assess* ***cure*** *to measure the effects of therapies in all future hand eczema trials?*						
not important		important but not critical		critically important, should be included in the core domain set		unable to score
1	2	3	4	5	6	
☐	☐	☐	☐	☐	☐	☐
Would you like to leave a comment or question? ______________________________						

**Chronicity**<br>This means if the hand eczema is lasting for a long time, which means longer than 3 months or at least two episodes in a year.<br>*How important is it to assess* ***chronicity*** *to measure the effects of therapies in all future hand eczema trials?*						
not important		important but not critical		critically important, should be included in the core domain set		unable to score
1	2	3	4	5	6	
☐	☐	☐	☐	☐	☐	☐
Would you like to leave a comment or question? ______________________________						

Additional suggestions

Would you like to suggest an additional domain or sub-domain?
If there is any domain or sub-domain that we may have missed, you may suggest to include them in the next round. Please suggest only domains or sub-domains that, in your opinion, are considered most important and should be included in all future trials to measure the effects of therapies for hand eczema. Please do not suggest specific outcome measurement instruments (such as HECSI).

____

Round 1: Conclusion

THANK YOU for having replied to this first round of the survey! We highly appreciate your support.

WHAT NEXT?
Within the next weeks [expected date], you will receive the invitation for the 2nd round via email with a NEW link to the survey. PLEASE BEAR WITH US!

In the **second round** the **results of the whole group of participants from the first round will be fed back to you and we will ask for a re-rating**. This offers you the chance to reconsider your personal rating. The goal of this procedure is that all participants finally reach an agreement about the most relevant outcomes.

In the second round, we are NOT voting again on items which already reached consensus 'in' (rated 'critical' by ≥ 80% of participants) or consensus 'out' (rated 'critical' by ≤ 50% of participants). Therefore, **it is very likely that the long list of candidate items will be reduced considerably after the first round.**

After the second round, there will be a consensus meeting (https://www.c3outcomes.org/events) in which we discuss the results of this eDelphi survey. Everybody who replies to both eDelphi rounds will be invited to the meeting, with the goal to agree on a complete set of core outcome domains. For those who cannot attend in person, there will be an online survey afterwards to confirm or refine the core domain set.

We reiterate that **anonymity of the responses is maintained. It is very important that the survey is COMPLETED in each round.** Otherwise, the reliability of the results could be compromised before the study is completed. If people drop out because they feel their opinions are in the minority, the final results will overestimate how much the sample of participants agreed on this topic.

Note also that **UPON COMPLETION of both rounds**, we would like to **ACKNOWLEDGE YOUR CONTRIBUTION** in the envisaged journal publication if desired. In round 2, you will have the option to indicate if you would like your name to be mentioned in the acknowledgments. We will not publish how the individual participants have voted.
Again, many thanks and we look forward to your feedback in the second round.

On behalf of the HECOS initiative: Henriette Rönsch - Andrea Bauer - Christian Apfelbacher

Round 2

Round 2: Introduction

Dear Participant,

In this second round, we ask you to finalize your opinion concerning [number not yet specified] overarching hand eczema domains, followed by [number not yet specified] more specific sub-domains. Which of these items should be core domains? In other words, which items should be used in **all** future hand eczema trials to measure the effects of treatments? You now have the option to change your first-round answers based on the answers of the other participants.

Please be aware that it is burdensome for patients as well as researchers to assess a large set of core domains during trials. We therefore ask you to vote 'include' only for domains and sub-domains that in your opinion are MOST important for the majority of patients with hand eczema.

We apply the following consensus criteria:

- **Consensus 'in'**: rated 'critically important' by ≥ 80% of participants
- **Consensus 'out'**: rated 'critically important' by ≤ 50% of participants
- No consensus: all other cases (rated 'critically important by more than 50% but less than 80% of participants)

Round 2: Questionnaire

<table>
<tr><td colspan="7">[Domain or sub-domain]
[explanation, if applicable]
How important is it to assess [domain or sub-domain] to measure the effects of therapies in all future hand eczema trials?</td></tr>
<tr><td colspan="2">not important</td><td colspan="2">important but not critical</td><td colspan="2">critically important, should be included in the core domain set</td><td>unable to score</td></tr>
<tr><td>1</td><td>2</td><td>3</td><td>4</td><td>5</td><td>6</td><td></td></tr>
<tr><td>☐</td><td>☐</td><td>☐</td><td>☐</td><td>☐</td><td>☐</td><td>☐</td></tr>
</table>

Round 2: Screenshot

HECOS: Development of a hand eczema core outcome set | Round 2 | Domains and sub-domains

DOMAINS | SIGNS OF HAND ECZEMA | SYMPTOMS OF HAND ECZEMA | HAND ECZEMA RELATED QUALITY OF LIFE | HAND ECZEMA CONTROL OVER TIME | PATIENT-REPORTED TREATMENT EXPERIENCE UNCLASSIFIED SUB-DOMAINS |

Please try to balance your own rating with the spirit of consensus and the patient perspective. In the figures, the left bar shows how many patients with hand eczema (or caregivers of a patient) found the outcome critically important. The right bar shows how many healthcare professionals and researchers found it critically important.

	not important		important but not critical		critically important, should be included in the core domain set		unable to score	Comment
	1	2	3	4	5	6		
**Domain 'patient-reported treatment experience'**<br>This is about how patients experience their overall hand-eczema treatment.<br>*How important is it to assess **the patient-reported treatment experience** to measure the effects of therapies in all future hand eczema trials?*	○	○	◉	○	○	○	○	🗨

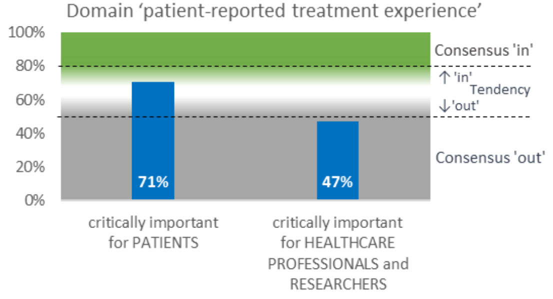

Round 2: Conclusion

Round 2: Additional consent

☐ I am willing to receive invitations to further projects of the Hand Eczema Core Outcome Set (HECOS) initiative. I have read and understood the information on data security [link to data security further invitations] and I grant permission to store and process my name and email address accordingly.

☐ I would like my contribution to this survey to be acknowledged in the envisaged journal publication. Please list my name in the acknowledgments section as follows:

- Patients/caregivers: Please enter your name and country of residence: _______
- Professional experts: Please enter your name and affiliation: ______

THANK YOU for completing our survey! We highly appreciate your support.

WHAT NEXT?
There will be an international meeting to finalize the consent or core domains for therapeutic hand eczema trials. We are going to invite everybody who have indicate their consent on the previous page and provide information concerning the meeting by email.

On behalf of the HECOS initiative: Henriette Rönsch - Andrea Bauer - Christian Apfelbacher
