# Supplementary material for: Consensus on core domains for hand eczema trials: Signs, symptoms, control and quality of life
Source: J Eur Acad Dermatol Venereol. 2025 Apr 25;39(9):1588–99. doi: 10.1111/jdv.20671 (PMC12376261; doi:10.1111/jdv.20671)

HECOS: Hand Eczema Core Outcome Set Consensus Meeting for therapeutic trials domains

September 3-4, 2024

The CHORD COUSIN Collaboration

A collaboration of international stakeholders interested in advancing outcome measurement for skin conditions.

Development of the core domain set (CDS)

Review of outcome domains in previous research

Consultation of experts

- Interviews with patients with hand eczema
- Online survey with professional experts of hand eczema

2-stage online survey (eDelphi)

- To reach consensus on a preliminary CDS
- To reach consensus on excluded (sub-)domains
- To list all controversial items
- Participants who completed both rounds: N = 132

Consensus meeting

- To discuss the preliminary CDS and excluded domains
- To reach consensus on controversial items

What happened in the online surveys

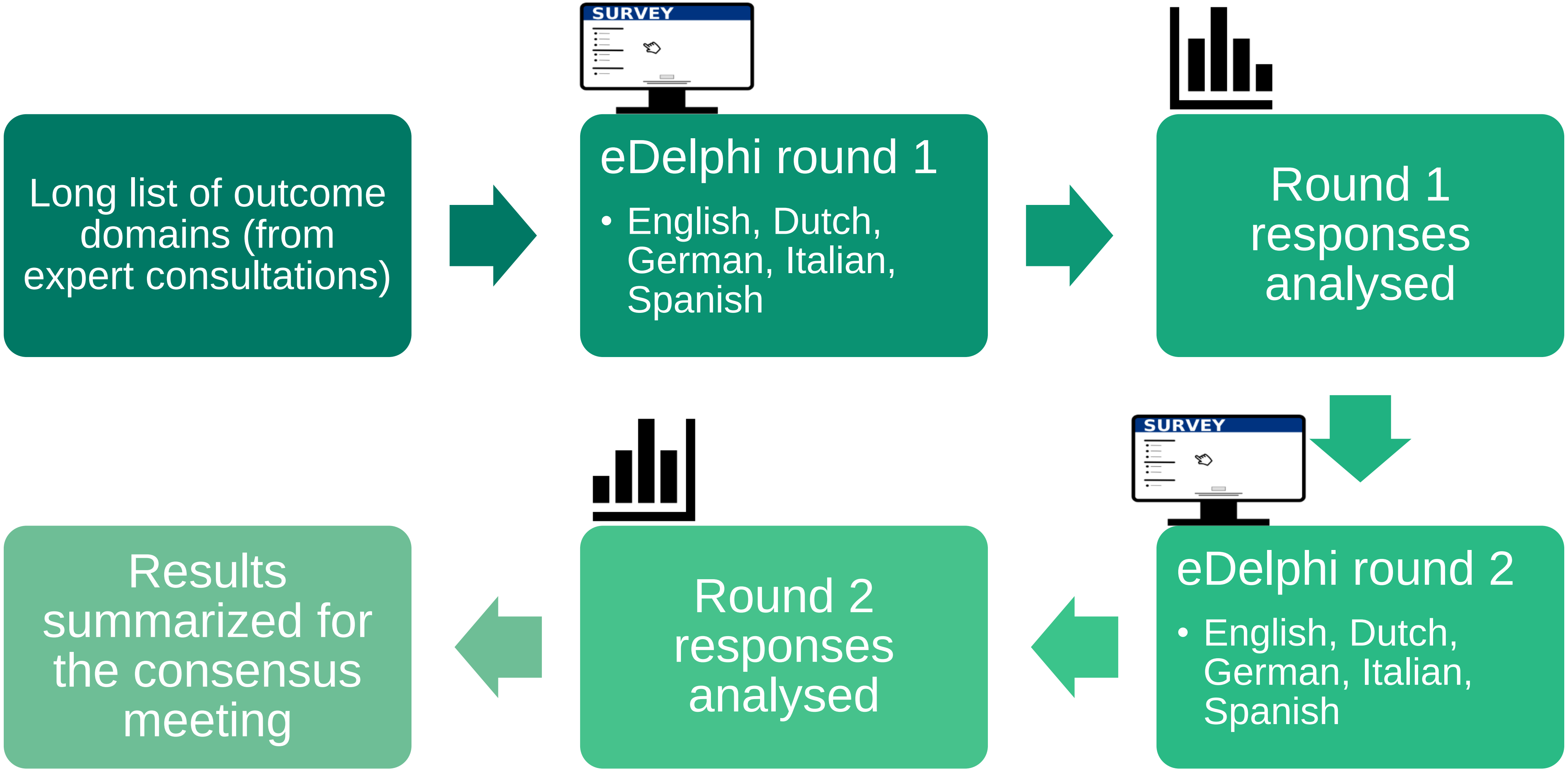

Results of the online survey (eDelphi):
Domains and sub-domains with consensus ‘in‘, consensus ‘out‘, or no consensus

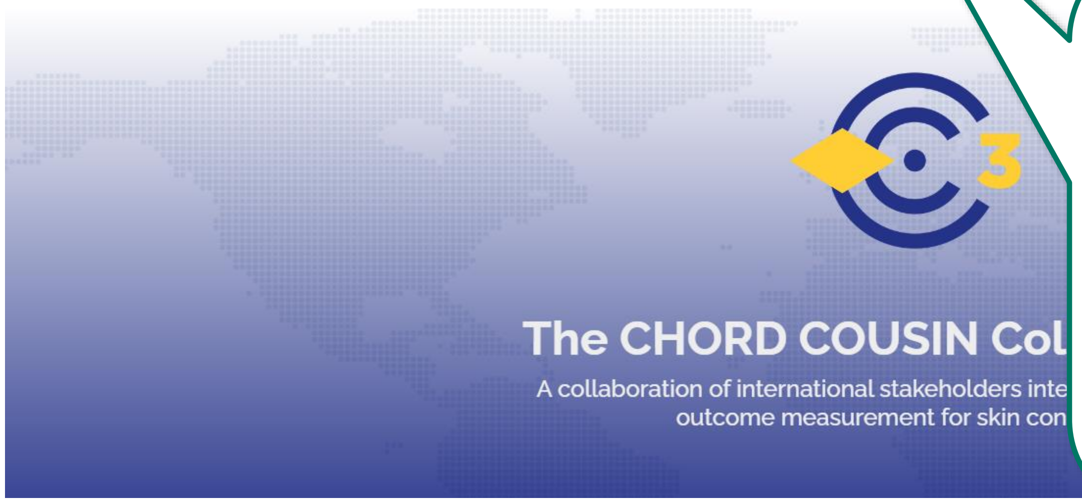

- The meeting‘s focus will be on (sub-)domains that have not yet reached consensus during the eDelphi.
- However, you will have the opportunity to discuss all included or excluded items.
- In case of objections (during the meeting), we are going to vote on controversial items again.

Preliminary consensus on domain level

Legend
included
excluded
no consensus

Signs of hand eczema	Symptoms of hand eczema	Hand eczema control over time
Hand eczema related quality of life	Patient-reported treatment experience	Skin barrier function

Sub-domains voted “in” – included in the preliminary core domain set

- **Signs of hand eczema**
  - Vesicles (blisters)
  - Fissures, rhagades (cracks)
  - Erythema
  - Infiltration
- **Symptoms of hand eczema**
  - Pruritus (itching)
- **Quality of life**
  - Physical hand functioning
  - Ability to work or study
  - Ability to take care of oneself or family
- **Hand eczema control over time**
  - Patient global assessment of treatment response
  - Number of flares in a given time
  - Area affected
- **Patient-reported treatment experience**
  - Treatment tolerability
- **Uncategorized sub-domains**
  - Cure
  - Chronicity

Sub-domains voted “**out**” – not included in the preliminary core domain set

- Signs of hand eczema
  - Dry skin
  - Nail changes
- Symptoms of hand eczema
  - Prickling
  - Stinging
  - Burning*
  - Sensitive skin*
  - Tight skin, impaired skin flexibility
- Quality of life
  - Extra efforts
  - Financial impact of hand eczema
  - Problem for loved ones
  - Not having to think about hands, having the mind free for other things
  - Conscious or unconscious, automatic scratching
  - Skin appearance, attractiveness of the skin, visibility of skin lesions
- Skin barrier function
  - Transepidermal water loss (TEWL)
- Hand eczema control over time
  - Number of unscheduled doctor visits related to hand eczema

* Burning & sensitive skin: critically important for > 70% of patients
(see below for detailed eDelphi results)

Meeting’s focus: Outcomes with no consensus

- **Signs**
  - Erosions, excoriation (scratch marks)
  - Lichenification (thickened hard skin)
  - Hyperkeratosis (rough and scaly patches of skin)
  - Desquamation/scaling
  - Oozing (clear fluid that comes out of the skin)
  - Edema (swelling)
  - Bleeding/crusting
- **Symptoms**
  - Aching/pain
- **QoL**
  - Emotional impact
  - Psychosocial impact
  - Sleep disturbances
  - Ability to practice leisure activities
- **Patient-reported treatment experience**
  - Treatment satisfaction
- **Control**
  - Period of time that is free of signs or symptoms of hand eczema
  - Global assessment of treatment response by medical staff
  - Use of additional medication or care products for the hands

Detailed results of the online survey (eDelphi)

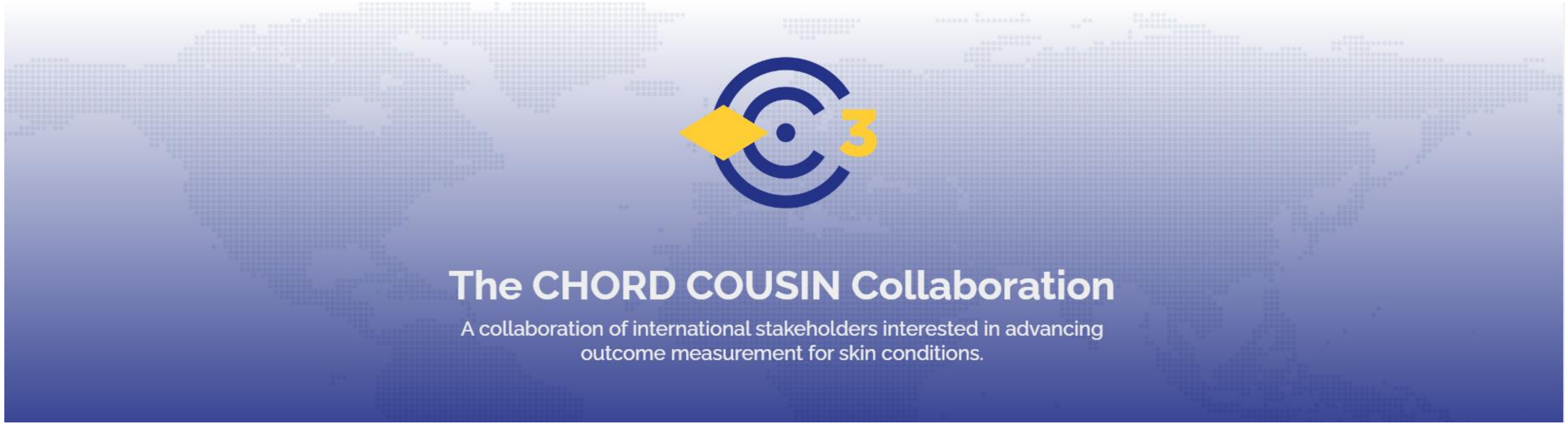

Included ‘signs’ sub-domains

1-2 not important

3-4 important but not critical

5-6 critically important, should be included in the core domain set

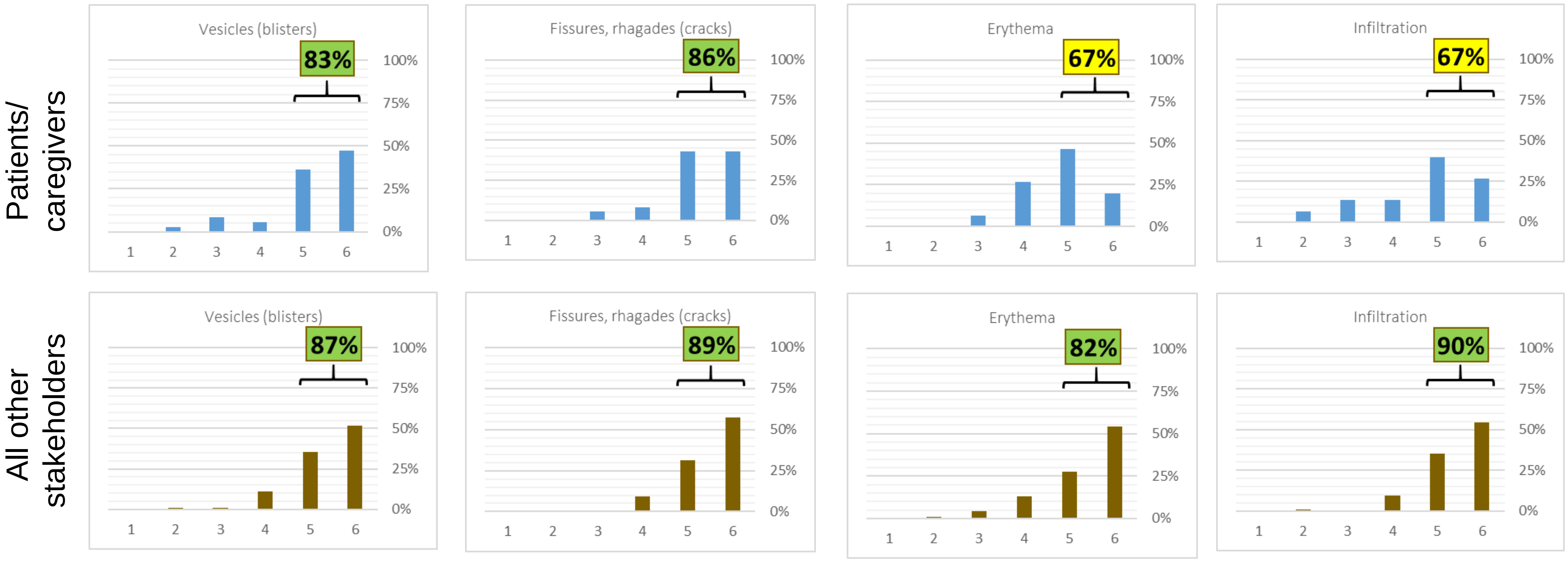

Excluded ‘signs’ sub-domains

Patients/ caregivers

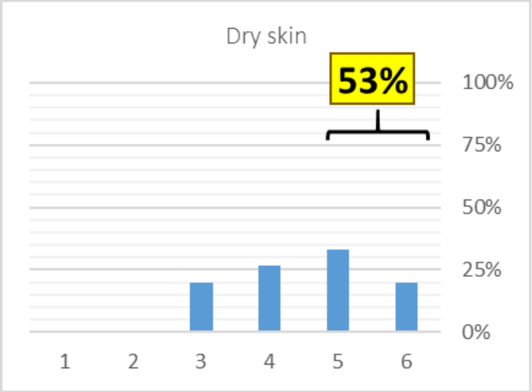

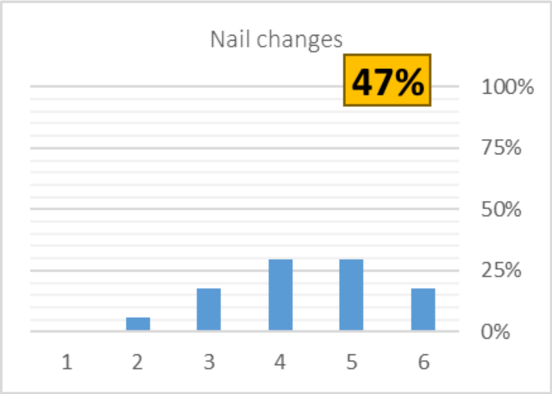

All other stakeholders

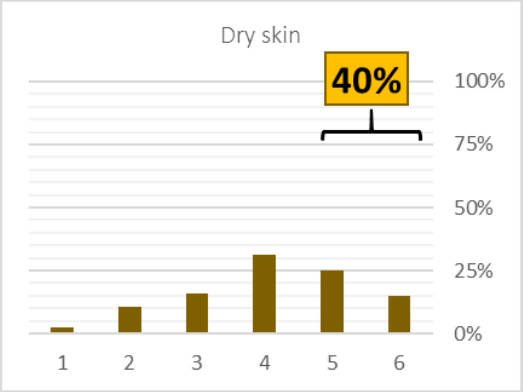

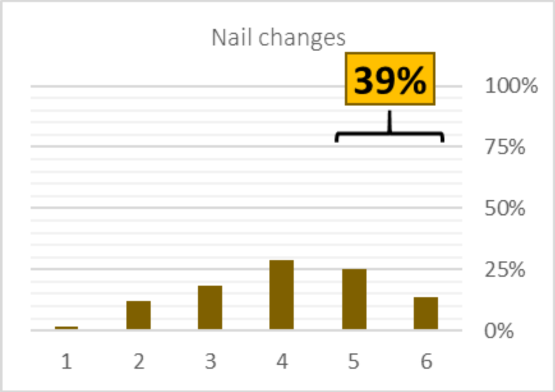

Included ‘symptoms’ sub-domains

Patients/ caregivers

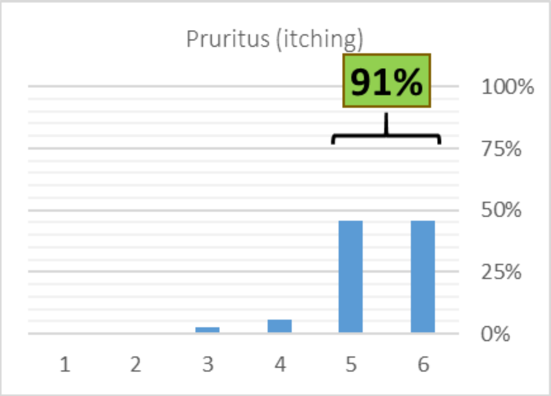

All other stakeholders

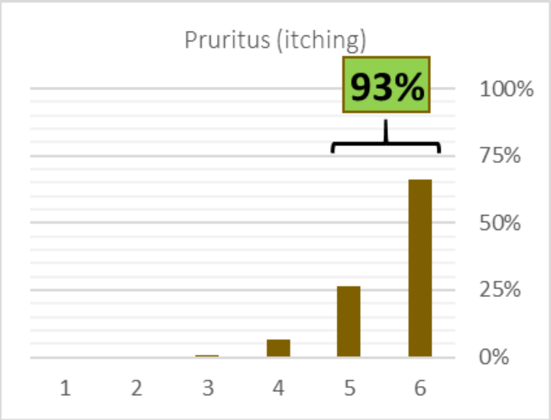

Excluded ‘signs’ sub-domains

Patients/caregivers

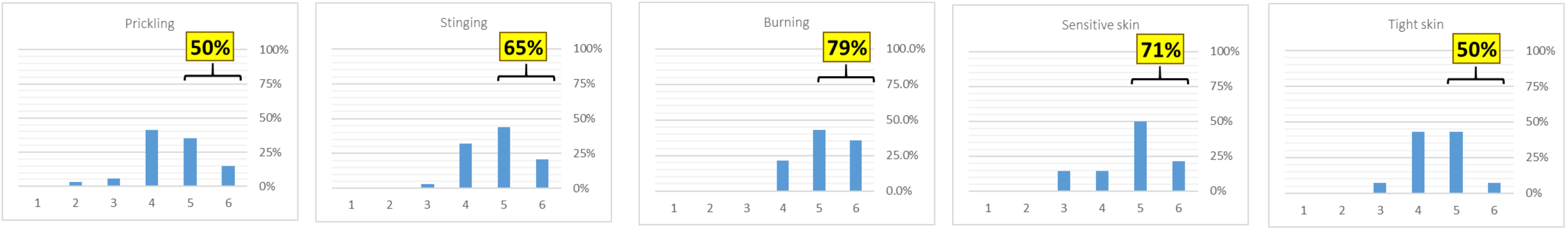

All other stakeholders

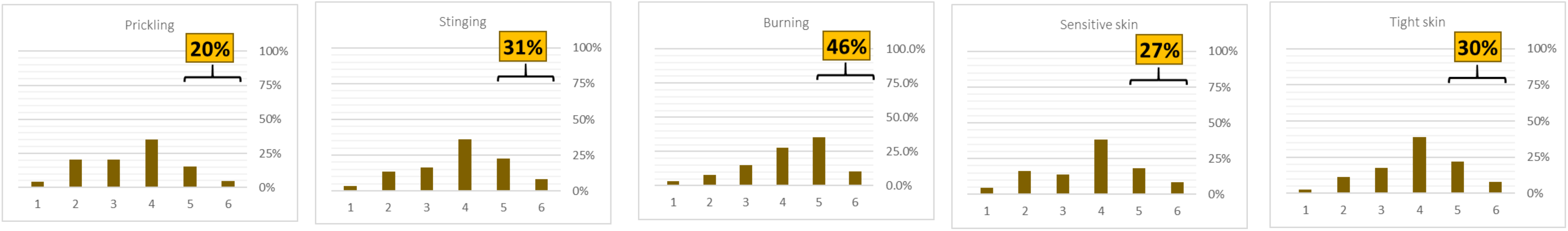

Included ‘QoL’ sub-domains

Patients/ caregivers

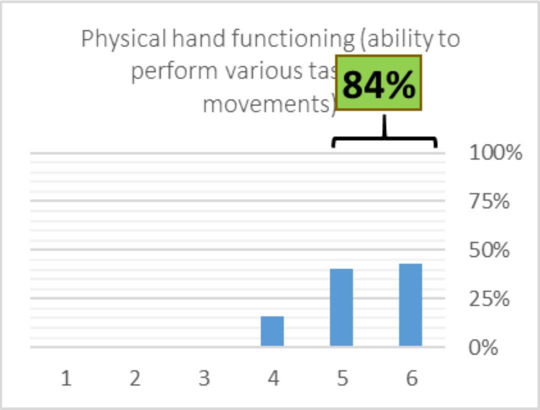

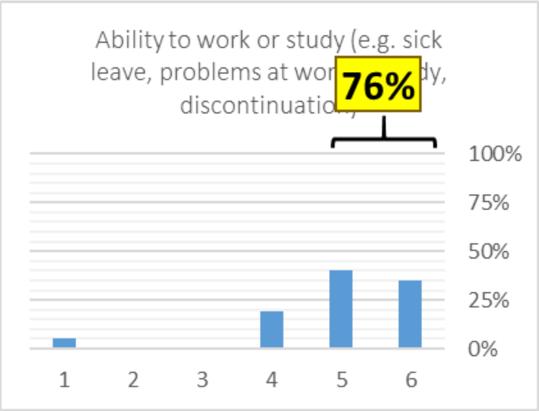

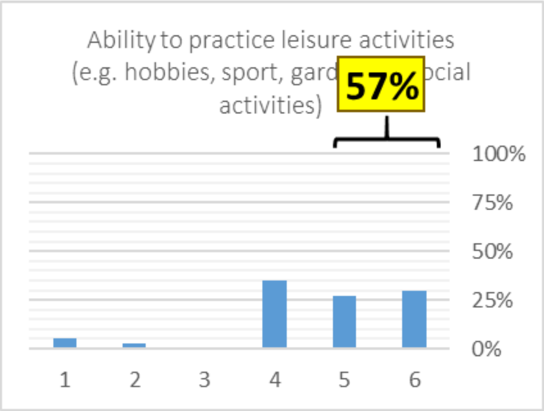

All other stakeholders

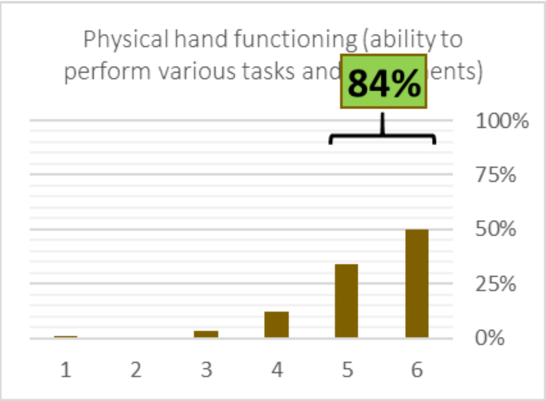

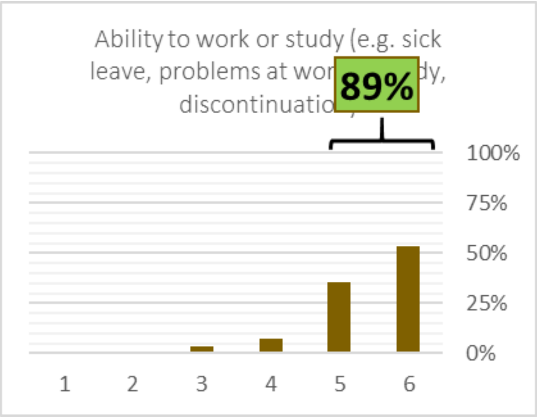

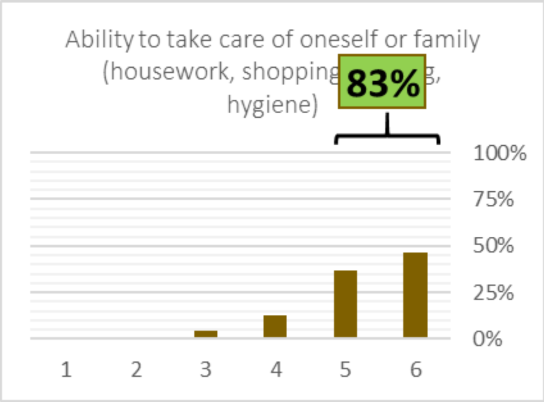

Excluded ‘QoL’ sub-domains

Patients/caregivers

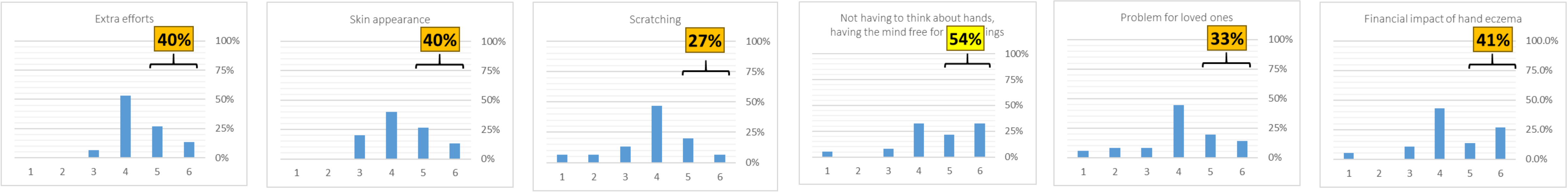

All other stakeholders

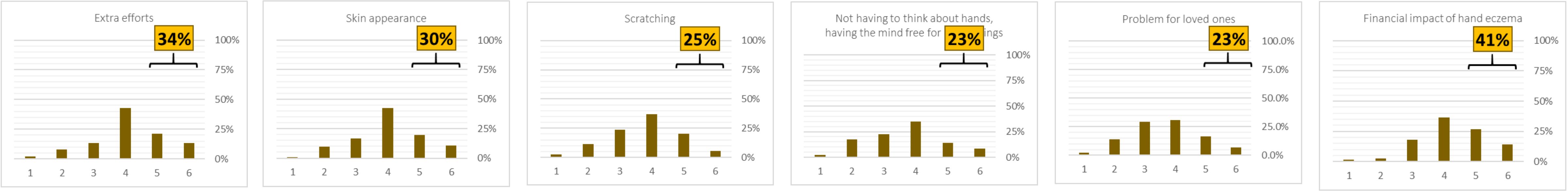

Included ‘control’ sub-domains

Patients/ caregivers

All other stakeholders

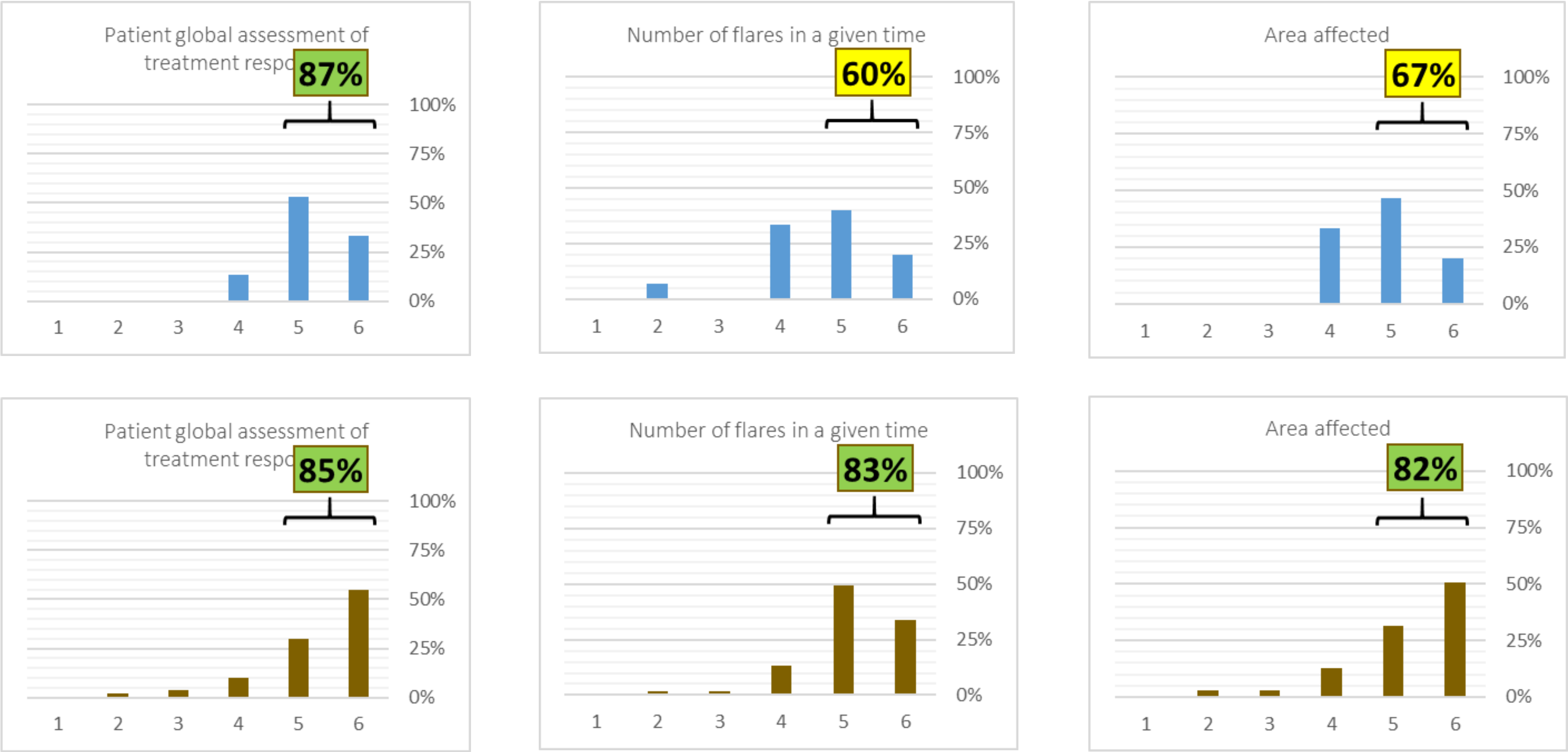

Excluded ‘control’ sub-domains

Patients/ caregivers

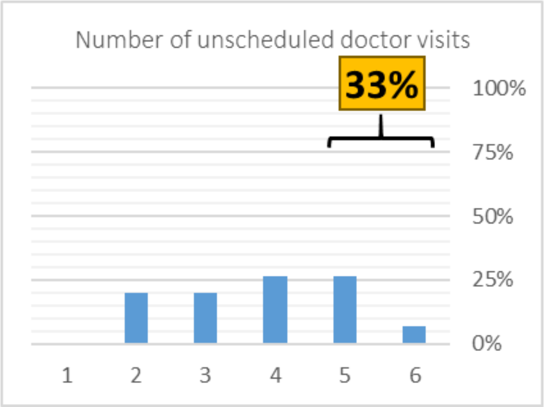

All other stakeholders

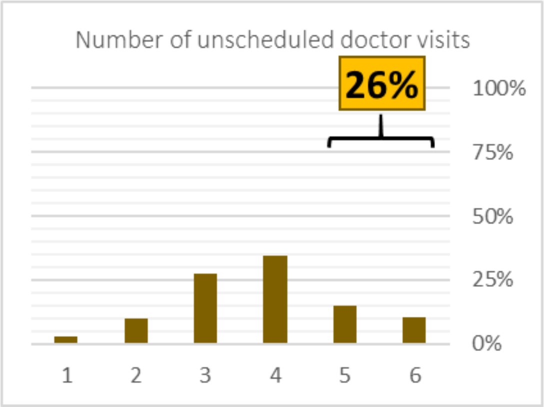

Included 'treatment experience' sub-domains

Patients/ caregivers

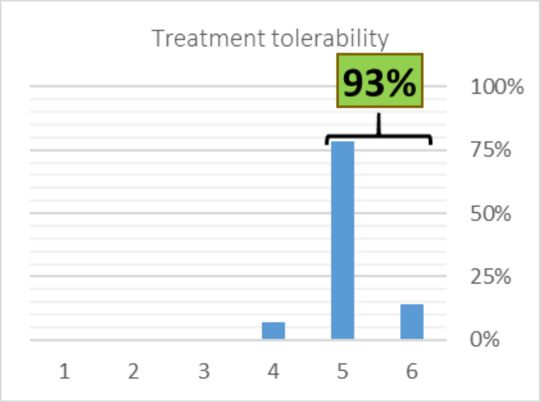

All other stakeholders

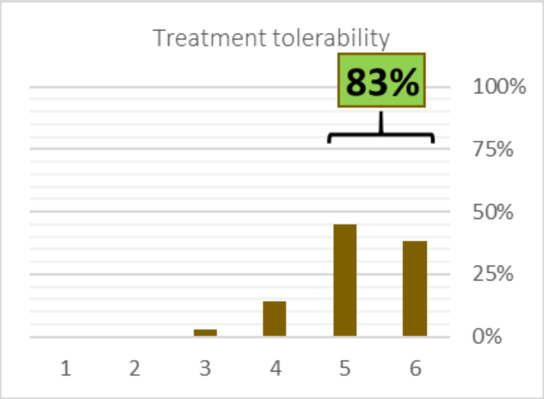

Excluded ‘skin barrier function’ sub-domains

Patients/ caregivers

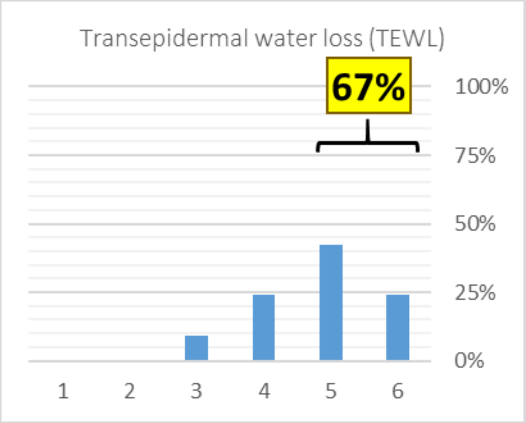

All other stakeholders

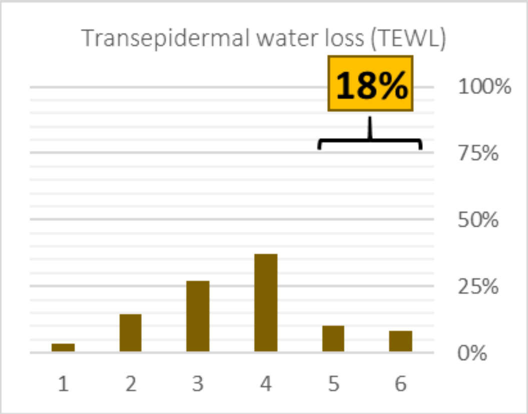

Included uncategorized sub-domains

Patients/ caregivers

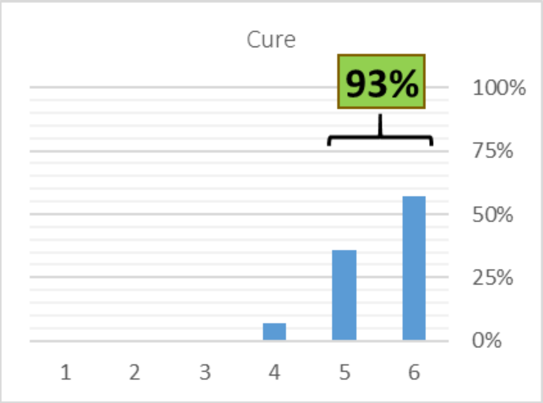

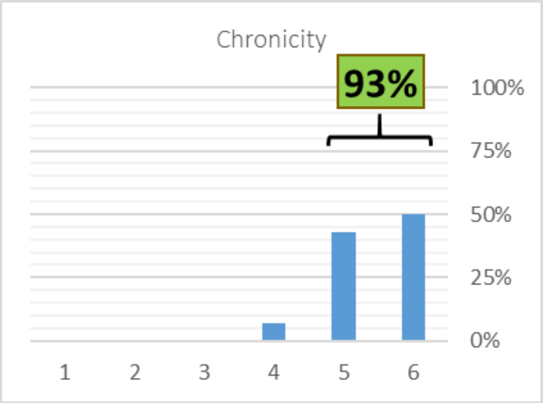

All other stakeholders

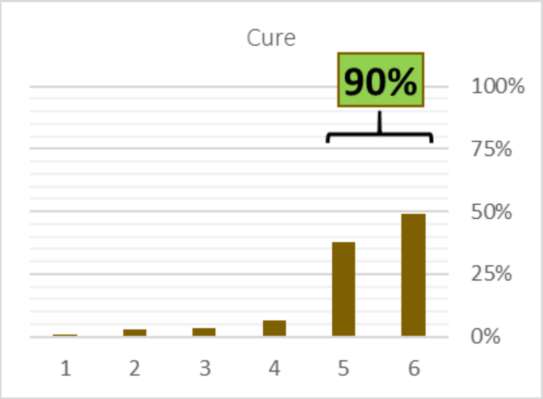

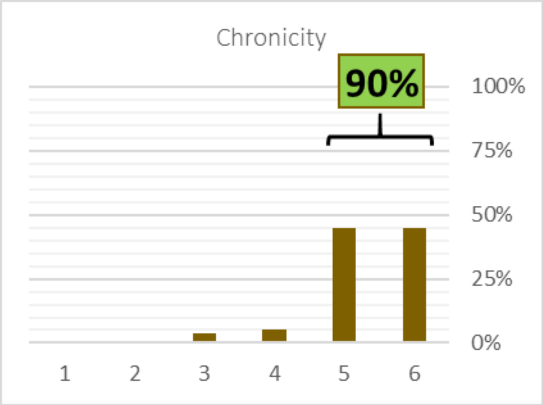

Included domains

Patients/
caregivers

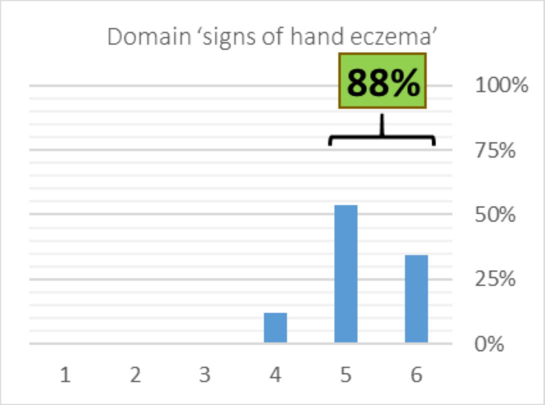

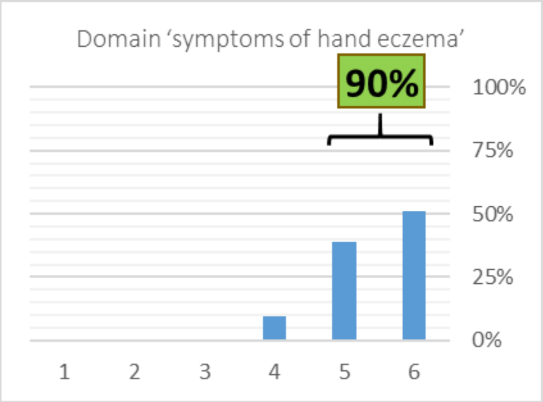

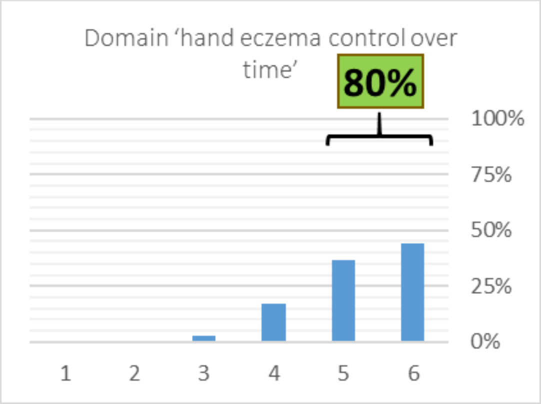

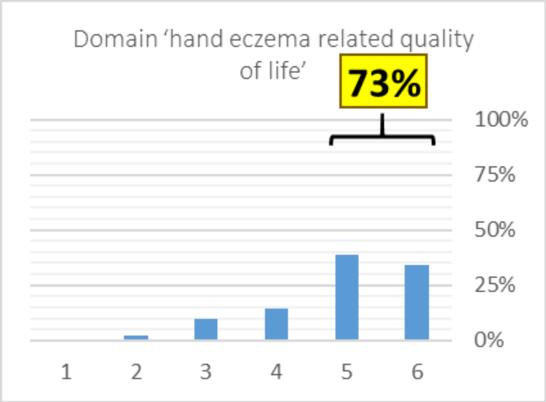

All other
stakeholders

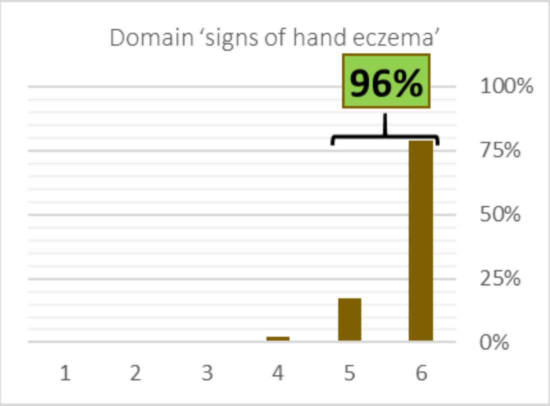

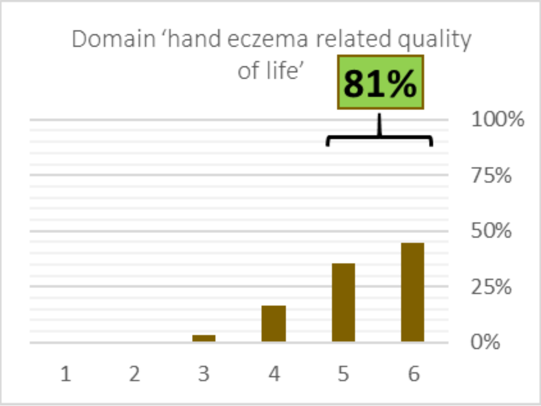

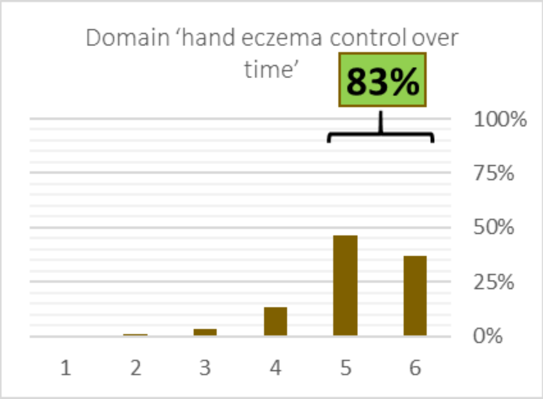

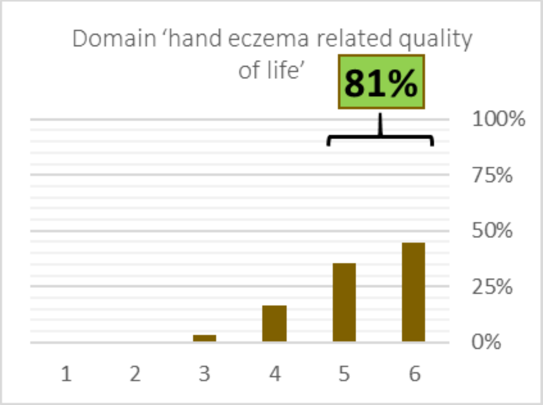

Excluded domains

Patients/ caregivers

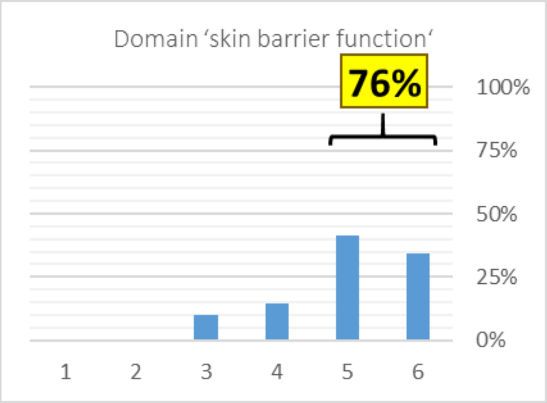

All other stakeholders

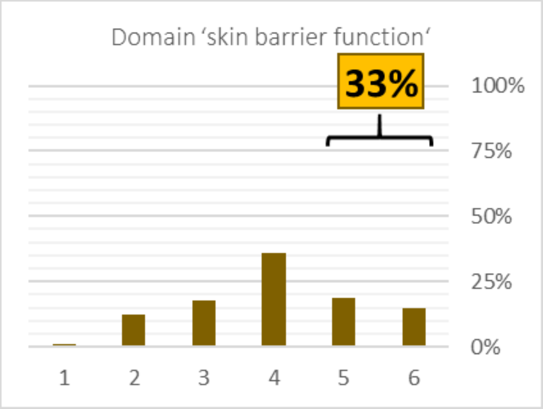

Supplement: Supplementary file 1 — Appendices S1‐S10 [file JDV-39-1588-s001.zip › jdv20671-sup-0002-AppendixS2.pdf]
