# Supplementary material for: Consensus on core domains for hand eczema trials: Signs, symptoms, control and quality of life
Source: J Eur Acad Dermatol Venereol. 2025 Apr 25;39(9):1588–99. doi: 10.1111/jdv.20671 (PMC12376261; doi:10.1111/jdv.20671)
Supplement: Supplementary file 1 — Appendices S1‐S10 [file JDV-39-1588-s001.zip › jdv20671-sup-0003-AppendixS3.pdf]

Glossary for the HECOS Meeting

Sources: HECOS (c3outcomes.org)

HECOS: Hand Eczema Core Outcome Set

Background Information

Hand eczema is a prevalent condition with significant personal and societal impacts. Despite numerous research efforts, the diversity of outcome measures in trials hampers comparability and the strength of clinical recommendations. The HECOS initiative seeks to address this by establishing a standardized core outcome set for both therapeutic and prevention trials, thereby improving the reliability and efficiency of hand eczema research.
The scope of our 2024 consensus meeting is core domains and sub-domains for therapeutic trials.

1. HECOS concepts and definitions

Hand **E**czema and occupational hand eczema:
It is a complex, multifactorial skin disease characterized by inflammation of the skin on the hands. Sings and symptoms include redness, itching, blistering, and scaling. It has a significant psychological and economic impact due to its high prevalence and chronic nature.
An occupational hand eczema is a form of hand eczema caused or exacerbated by workplace exposures. It is one of the most common occupational diseases, comprising 40% of all such diseases in industrialized nations.

Core **O**utcome **S**et (COS)
It is an agreed standardized set of outcomes that should be measured and reported in all clinical trials for a specific condition. In the context of HECOS, there are two COS: one for therapeutic trials and one for prevention trials.

2. Key Terms

Clinical trials
Research studies that test how well new medical approaches work in people. They help determine if treatments are safe and effective.

Core domain set
A set of domains constituting the minimum that should be measured in a specified field of research. Unlike core outcome sets, core domain sets do not (yet) specify outcome measurement instruments.

Consensus
General agreement among a group. In developing a COS for hand eczema, consensus means that most stakeholders, like doctors, patients, and researchers, agree on which outcomes are most important to measure. For example, they might all agree that measuring the severity of itching, the frequency of flare-ups, and the impact on daily activities are crucial outcomes.

Consensus process
A methodological approach to achieve agreement among experts and stakeholders on the core outcome set. This often involves multiple rounds of surveys and meetings to discuss and refine the candidate domains and sub-domains.

Efficacy/effectiveness
How well a treatment works under controlled conditions, like in a clinical trial. Efficacy outcomes for hand eczema might include how much a treatment reduces redness or itching. There is a subtle difference between efficacy and effectiveness, but this is not relevant for HECOS.

Long List
A comprehensive list of candidate outcome domains and sub-domains generated through systematic reviews, expert input, and stakeholder consultations. This list serves as the starting point for the consensus process.

Outcome
A result or effect of a treatment or medical condition. For hand eczema, an outcome could include how severe a specific symptom is, how often flare-ups occur, and how the hand eczema affects a specific daily activity.

Outcome domains
Broad categories of outcomes that are important for assessing the effectiveness of interventions in clinical trials. Examples include symptoms, quality of life, and treatment side effects.

Outcome measurement instrument
An instrument that is or could be applied to measure a domain, such as a questionnaire or technical device.

Patient-Reported Outcome (PRO)
Information about a patient's health directly from the patient, without interpretation by doctors or others. For hand eczema, this could include how itchy their skin feels or how the condition affects their daily life.

Relevance
How meaningful and significant an outcome is to patients and other stakeholders. For hand eczema, an outcome is relevant if it genuinely reflects what matters most to those affected by the condition.

Sub-domains
Specific aspects within an outcome domain. For example, within the domain of symptoms, sub-domains might include itching and pain.

Therapeutic hand eczema trials
Clinical studies aimed at evaluating the effectiveness of interventions to treat existing hand eczema. These trials focus on a variety of outcomes, such as alleviating symptoms, reducing recurrence, and improving the quality of life of patients.

Long List of Candidate Core Outcome Domains and Sub-Domains for Therapeutic Hand Eczema Trials

1. Signs of hand eczema
2. Symptoms of hand eczema
3. Hand eczema related quality of life
4. Skin barrier function
5. Patient-reported treatment experience
6. Hand eczema control over time
7. Unclassified sub-domains

1. Domain "signs of hand eczema"

This means all the changes in the skin that a doctor, patient, or another person can look at during an examination.

Sub-domains within the domain "signs of hand eczema"

1.1. Erythema (redness)

1.2. Infiltration (elevated skin): papules, patches, plaques, nodules
This refers to the skin getting thicker, causing raised changes that you can feel, either with or without thick scaling. This thickening usually happens in specific areas, forming different types of bumps.

1.3. Edema (swelling)
This means that extra fluid builds up in the skin, making it puffy.

1.4. Vesicles (blisters)

1.5. Erosions, excoriation (scratch marks)

1.6. Fissures, rhagades (cracks)

1.7. Oozing (clear fluid that comes out of the skin)

1.8. Bleeding/crusting

1.9. Desquamation (when the outer layer of the skin peels off or flakes)

1.10. Lichenification (thickened hard skin)
This means areas of the skin that are thick and hard from constantly scratching or inflammation. This is different from "keratosis," which are areas of the skin that are thick and scaly.

1.11. Keratosis (rough and scaly patches of skin)

1.12. Dry skin

1.13. Nail changes

2. **Domain "symptoms of hand eczema"**
   This means all skin complaints that only the patient can feel and describe.

Sub-domains within the domain "symptoms of hand eczema"

2.1. Aching/pain

2.2. Prickling

2.3. Stinging

2.4. Burning

2.5. Pruritus (itching)

2.6. Sensitive skin
This means when the skin feels uncomfortable—like stinging, burning, pain, itching, or tingling—when it is exposed to things like heat, cold, cosmetics, or water, even though these things usually should not make the skin feel that way.

2.7. Tight skin, impaired skin flexibility

3. **Domain "Hand eczema related quality of life"**
   This means all aspects of life that are affected by hand eczema.

Sub-domains within the domain "hand eczema related quality of life"

3.1. Physical hand functioning (ability to perform various tasks and movements)
This is about how well the hands can perform various tasks and movements. It includes actions like gripping, holding, and moving the fingers.

3.2. Ability to work or study (e.g. sick leave, problems at work or study, discontinuation)

3.3. Ability to take care of oneself or family (housework, shopping, bathing, hygiene)

3.4. Ability to practice leisure activities (e.g. hobbies, sport, gardening, social activities)

3.5. Extra efforts
This refers to any additional work patients have to take in order to care for the hand eczema. This includes having to attend doctor appointments, needing to use gloves, and having to spend time applying lotions and creams.

3.6. Emotional impact (well-being, mood, unrest, depression, anger, annoyance, frustration, humiliation, shame, fears, worries, helplessness, acceptance, hope in treatment)

3.7. Psychosocial impact (e.g. concerning shaking hands, impairment of social contacts, relationships or sexual life, stigmatization, difficulties showing affection, self-consciousness, staying at home because of hand eczema, influence on clothing, withdrawal from social life)

3.8. Financial impact of hand eczema
This is about how the treatment a person gets can help them spend less money on things like over-the-counter products, extra treatments, doctor visits, travel, and not being able to work (which is lost income).

3.9. Problem for loved ones
This refers to any impact that the hand eczema has on patients' loved ones.

3.10. Not having to think about hands, having the mind free for other things

3.11. Conscious or unconscious, automatic scratching

3.12. Sleep disturbances

3.13. Skin appearance, attractiveness of the skin, visibility of skin lesions

4. Domain "skin barrier function"
This is about whether that protective layer of the skin is working well or not.

Sub-domains within the domains "skin barrier function"

4.1. Transepidermal water loss (TEWL)
This is when water is lost through the skin, like when it evaporates. If the skin is irritated due to hand eczema, it does not protect well, so more water is lost. Researchers measure TEWL with a special tool, and a high value might mean that the skin is damaged.

5. Domain "Patient-reported treatment experience"
This is about how patients experience their overall hand-eczema treatment.

Sub-domains within the domain "patient-reported treatment experience"

5.1. Treatment satisfaction
This means how satisfied someone is regarding the cosmetic and/or functional results of treatment.

5.2. Treatment tolerability
Treatment tolerability means how well a person can handle and endure a particular treatment. It is about how comfortable or manageable the treatment is for the individual.

6. Domain "hand eczema control over time"
This is all about how well-controlled the hand eczema is over time. By regularly checking things like how severe it is or how often it flares up, we can figure out if hand eczema is being well controlled over time.

Sub-domains within the domain "hand eczema control over time"

6.1. Patient global assessment of treatment response
Global assessment of treatment response means looking at how well an entire treatment is working overall. It involves evaluating if the treatment is helping, making things better, or if there are still issues.

6.2. Period of time that is free of signs or symptoms of hand eczema

6.3. Number of flares in a given time (e.g. number of flares in the past three months)
This means that it is recorded how often hand eczema gets worse during a given period of time.

6.4. Area affected
This means how large of an area is affected by the condition. For example, it may be only one thumb or the entire hand.

6.5. Global assessment of treatment response by medical staff

6.6. Number of unscheduled doctor visits related to hand eczema
In a study that investigates how well a certain medication/treatment works, there will be regular study visits. Unscheduled doctor visits means how often a patient needs to visit a physician because of the hand eczema, in addition to the regular study visits or other regular visits.

6.7. Use of additional medication or care products for the hands
In a study, that investigates how well a certain medication/treatment works; patients may still need additional medication, such as pills or creams, or moisturisers. This may show how well the study medication works. For example, an effective study medication may allow a patient to use less cortisone.

7. Unclassified sub-domains

7.1. Cure
This means absence from signs and symptoms, normal looking skin of the hands.

7.2. Chronicity
This means if the hand eczema lasts for a long time, specifically longer than 3 months or at least two episodes in a year.
