# Supplementary material for: Consensus on core domains for hand eczema trials: Signs, symptoms, control and quality of life
Source: J Eur Acad Dermatol Venereol. 2025 Apr 25;39(9):1588–99. doi: 10.1111/jdv.20671 (PMC12376261; doi:10.1111/jdv.20671)
Supplement: Supplementary file 1 — Appendices S1‐S10 [file JDV-39-1588-s001.zip › jdv20671-sup-0005-AppendixS6.pdf]

HECOS eDelphi therapeutic domains – Suggestions from participants (round 1)

May 13, 2024

Suggestions of additional domains and sub-domains which ARE INCLUDED in round 2

- Global assessment of treatment response by medical staff
- Number of unscheduled doctor visits related to hand eczema
- Use of additional medication or care products for the hands

Suggestions of additional domains and sub-domains which ARE NOT INCLUDED in round 2

In round 2 of the eDelphi, we added only items that can be measured to determine how well a therapy works. The following groups of suggestions were not added because they are not strictly therapeutic effectiveness domains/sub-domains but related concepts:

- Comorbidity and patient baseline characteristics, such as atopic skin status or psoriasis.
- Complications, such as warts or other contagious skin changes, because changes concerning these complications do not directly measure the effectiveness of a hand eczema treatment.
- Presence or absence of changes on the feet or any other body part, because changes on other parts of the body are beyond the defined scope of HECOS.
- Properties of the therapy/medicine if they do not indicate how effective the therapy is, such as costs or greasiness.
- Patient work conditions and behaviour, such as protective measures or lifestyle factors.
- Advice for diagnosis, treatment, or management of the hand eczema.
- Specific outcome measurement instruments (*how* to measure), such as corneometry. We are going to address outcome measurement instruments in the next phase of HECOS.
- Methodological advice for outcome measurements or trial conduction, such as washout phases or assessment of aggravating factors.
- Time frame of healing, because it cannot directly measure treatment effectiveness and is a metric of another domain, healing.
- Presenteeism (working while having hand eczema, working while it would have been better to be on sickness absence), because it cannot directly measure treatment effectiveness and relies on other domains to define when sick leave is indicated.
- Involvement of the volar vs dorsal surfaces of the hand or digits, because it cannot show how effective a treatment is. Instead, it can be a valuable variable for stratification.

The following suggestions were not added because they belong to the domain 'skin barrier' function, for which eDelphi participants already reached consensus 'out' after round 1:

- Skin pH
- Skin dryness (corneometry)
- Natural Moisturizing Faktor (Ramanspectroscopy)

Finally, suggestions that were already covered by the survey were not added, such as 'sleep loss' (covered by 'sleep disturbances') and 'patient-reported global severity of hand eczema' (covered by 'global assessment of treatment response').
