# Supplementary material for: Consensus on core domains for hand eczema trials: Signs, symptoms, control and quality of life
Source: J Eur Acad Dermatol Venereol. 2025 Apr 25;39(9):1588–99. doi: 10.1111/jdv.20671 (PMC12376261; doi:10.1111/jdv.20671)
Supplement: Supplementary file 1 — Appendices S1‐S10 [file JDV-39-1588-s001.zip › jdv20671-sup-0006-AppendixS7.pdf]

HECOS eDelphi therapeutic domains – Comments from participants (rounds 1&2)

August 23, 2024

Signs

Erythema (redness) / edema

- Assessment of erythema is problematic in patients with dark skin.
- perhaps the most difficult sign to assess and record. where is the border between erythema and nonerythema, how do we assess the degree and extension, changes in it??However, in darker skin tones, erythema may not be considered a core eczema sign.

Symptoms

- 'Prickling, stinging, and burning' should be combined.
    - Each of these sub-domains reached consensus 'out'. However, 'burning' received high ratings from patients; we therefore present this item again.
- In Denmark the patients don't say 'burning'. They would say 'stinging' or 'painful'.

Dry skin

- I have difficulties in deciding either this is 4 or 5 in my decision. Of course it is important, but dry skin itself, easy to treat with emollient only is so common so it gives noise to the system

Sensitive skin

- It seems of little relevance to me in hand eczema
- in my previous comments I highlighted what I believe about subjective signs. One could believe that is is possible to create two domains for judgement, one for subjective feelings that exist without extern stimulous and one upon extern stimulous. But to call it sensitive skin would be a mistake I believe. Sensitive skin is a tricky word constructed by cosmetic industry and people claim sensitive skin because they are such individuals who need care, hugs and acceptance. It is an impossible word to work with
- Interesting. I never ask about 'sensitivity' and it is only rarely that the patients use that term. I hear it from patients with atopic dermatitis but not with hand eczema.

Tight skin, impaired skin flexibility

- An objective measure of disability: Do you have pain or disability when touching the hypotener eminence with the ball of your third finger? or Do you have inability or pain when touching the pad of the 5th finger with the pad of the first finger?
- perhaps if we create and evaulate a multitranslation model for this domain. Interesting and perhaps less studied sign but it could be of interest
- Those with severe hand eczema do have problems with skin flexibility. But it is not so often in the clinic. However this is a trial setting. So, relevant I think.

QoL

Physical hand functioning (ability to perform various tasks and movements)

- it would be great, we have too little objective measurements but it would limitate the number of patients involved

Ability to work or study (e.g. sick leave, problems at work or study, discontinuation)

- This question is important, but could be included in the question about the functionality of the hands in genera

Ability to take care of oneself or family (housework, shopping, bathing, hygiene)

- This question is important, but could be included in the question about the functionality of the hands in general

Extra efforts

- very good idea of these "softer" values, which are very important but difficult to know and report without adequate questions
- Extra efforts: The patients spend extra time to take care of the hand eczema, this time could be useful to do other things with family or friends.
- The additional effort of a therapy may contribute less to the evaluation of the effect of a therapy for hand eczema, but should perhaps be taken into account when evaluating the therapy as a whole.

Financial impact of hand eczema

- This is a rather vague criterion that may greatly vary from country to country, but also between individuals.

Conscious or unconscious, automatic scratching

- Here the question of itching is more important.
- I don’t think the patients can answer the question about conscious/unconscious/automatic scratching precisely. Especially not the unconscious scratching.

Skin barrier function

- The domain ‘skin barrier function‘ is important but difficult to assess and not crucial for every therapeutic hand eczema trial.
- From scientific point of view it is a very important outcome but if it is applied to all studies it will diminish the number of patients involved, as it requires some kind of instrumentation, even if tape stripping would be a good alternative which is easy. So, depending on the method chosen it is in between crucial for a GOOD study, but maybe can considered with lower importance for the big number of inclusion
- It is highly difficult to assess the skin barrier function with e.g. TEWL or pH or conductance, as not only the lesional skin but also non lesional skin is affected. Furthermore, the hands are difficult to assess physically due to a irregular surface which results in inconclusive data.
- If a validated and reliable testing/determination of the skin barrier function is possible: The significance of the skin barrier function depends heavily on the study aim or the collective examined

Transepidermal water loss (TEWL)

- Never seen a patient come and ask me to cure his/her elevated TEWL.

- It would greatly limit the trials.
- as I mentioned in the top domains, the use of TEWL (besides that is requires expertese to perform it correctly, difficult to know where to measure on a hand with so different varity of symptoms) makes the number of participants MUCH lower. Consider tape analysis for NMF or similiar measures)
- It is a great non invasive skin barrier assessment, however, also linked to increased uncertainty when interpreting the data, as TEWL is affected by several external as well as internal factors. Owing to the irregular surface of the hands, I would always interpret the data with caution.
- Very variable, Requires such a special setting (se room, no showering, etc.)

Hand eczema control over time

- In my opinion, this is more of a holistic outcome that could possibly replace signs and symptoms.
- perhaps the most important and often underreported domain. Many therapies can give short term advantages, but cannot be applied in the long run. Hand eczema is a very chronic disease, without long lasting effects we have no therapy success

Number of flares in a given time (e.g. number of flares in the past three months)

- Interesting to capture however there needs to be a consistent definition of "flare"

Area affected

- Apart from the % of the area affected it may be very important to have specific areas (fingertips, fingers) ... it may gave worst impact on functionality than the dorsum of the hands the area is very important in many ways. Previous studies shown that the area affected is the most important long time prognostic factor. But also it belongs to the evaluation of severity too
- The size of the affected area does not always reflect the extent of the restriction and the severity of the hand eczema (e.g. in the case of pulpitis sicca).
- HECSI at minimum, which should be generally sufficient, but information from HESCI still has limitations for fully understanding area affected.

Patient global assessment of treatment response

- For how long? And how often?

Other

Cure

- How to define and measure it? E.g., how to define what is "normal" and what is not? Ask the patient "do you feel cured"?
- I would avoid the term “cure” and instead speak of “Erscheinungsfreiheit” [~ clear skin] or “remission”.
- Only if a treatment aimed to be curative through MoA and would need agreement on duration of remission for the treatment to be curative, e.g. 5 years?
- I don’t exactly know what ‘cure’ implies? By what method? One can never say cured for condition like this that is propensity.

- I am not sure that ‘cure’ is the best term to use for a chronic, relapsing condition. I understand this to be viewed more as complete resolution as a marker of treatment success and have scored accordingly.

Chronicity

- Does "chronicity" depend on the treatment or on the individual predisposition and exposures of the patient? Wouldn't it be more accurate to count number and duration of "relapses" instead?
- I don't quite understand the concept of ‘chronicity’ as an outcome measure. Chronicity as I understand it, is determined by the nature of a disease, or - on an individual level - by a patient's past history (e.g. has the present disease lasted for more than 3 months or not?). If one looks into further course of the disease, perhaps the time lapse to a partial or total cure would be more adequate outcome measure? Or the number of relapses in a given follow-up period?
