# Supplementary material for: Consensus on core domains for hand eczema trials: Signs, symptoms, control and quality of life
Source: J Eur Acad Dermatol Venereol. 2025 Apr 25;39(9):1588–99. doi: 10.1111/jdv.20671 (PMC12376261; doi:10.1111/jdv.20671)
Supplement: Supplementary file 1 — Appendices S1‐S10 [file JDV-39-1588-s001.zip › jdv20671-sup-0007-AppendixS8.pdf]

# HECOS eDelphi therapeutic domains – Comments from participants (round 1)

May 13, 2024

Many thanks to everybody who provided rationales for their ratings. This makes it easier for us to interpret your rating and will be considered in future decisions, especially in the face-to-face consensus meeting: https://www.c3outcomes.org/events

In addition to providing rationales, participants enriched the core outcome set development process with further remarks and ideas. Please find below a summary of all participant comments along with our replies.

## Summarised comments and replies

- [Comment:] It is important to focus on domains and sub-domains that are crucial and highly relevant for patients. Most items presented in this survey should be rated 1-4 (‘not important’ or ‘important but not critical’), otherwise it will be a burden for future study participants.
    - [Reply:] Exactly! We ask all participants to be as selective as possible and give a rating of 5-6 (‘critically important’) only to the most important domains and sub-domains.
- The broad domains like ‘signs of hand eczema’ or ‘hand eczema related quality of life’ are defined too broadly.
    - The survey’s first page was intended to display all broad outcome domains that might be relevant for therapeutic hand eczema research. The goal was to reach consensus whether or not domains such as ‘symptoms’ are essential at all. The following pages included sub-domains for each domain so that consensus can also be reached on a more specific level.
- Participants provided advice, thoughts, concerns and questions concerning measurement details, for example concerning timing.
    - We will take these comments into account for the next phase of HECOS, when we seek consensus about outcome measurement instruments (*how* to measure).
- Participants provided advice and thoughts about patient-centred healthcare.
    - We will try to share this with other clinicians.
- Please make the next survey mobile friendly.
    - We will do our best for the next survey. However, we cannot change the format for this survey’s round 2.
- The domain ‘skin barrier function‘ is important but difficult to assess and not crucial for every therapeutic hand eczema trial.
    - In accordance with these comments, the participants reached consensus ‘out’ for this domain.
- ‘Prickling, stinging, and burning’ should be combined.
    - Each of these sub-domains reached consensus ‘out’. However, ‘burning’ received high ratings from patients; we therefore present this item again.
- Extra efforts: very good idea of these "softer" values, which are very important but difficult to know and report without adequate questions
    - Thank you! This domain was suggested by the patients who participated in the HECOS interview study. If there is consensus to evaluate ‘extra efforts’ of hand eczema as part of the core outcome set, the HECOS initiative will identify or develop adequate ways

to assess it (as part of the next HECOS phase: development/consensus on core measurement instruments for the core domains; *how* to measure)

- The sub-domain 'desquamation (when the outer layer of the skin peels off or flakes)' is about scaling.
    - We have amended the name: 'desquamation/**scaling** (when the outer layer of the skin peels off or flakes)'
- Keratosis (rough and scaly patches of skin): Better to talk about hyperkeratosis than keratosis, it can be misleading
    - Right, we have corrected this.
- Keratosis could be combined with desquamation
    - We will keep this in mind for the consensus meeting. (https://www.c3outcomes.org/events)
- I would avoid the term "cure" and instead speak of "clear skin" or "remission".
    - This remark is substantiated; our definition and wording for this item may need revision. However, it is impractical and confusing to make a rather big adjustment like this between the eDelphi rounds. We will keep this in mind for the consensus meeting.
