# Supplementary material for: Consensus on core domains for hand eczema trials: Signs, symptoms, control and quality of life
Source: J Eur Acad Dermatol Venereol. 2025 Apr 25;39(9):1588–99. doi: 10.1111/jdv.20671 (PMC12376261; doi:10.1111/jdv.20671)
Supplement: Supplementary file 1 — Appendices S1‐S10 [file JDV-39-1588-s001.zip › jdv20671-sup-0009-AppendixS10.docx]

Table 4: Partial re-evaluation of eDelphi consensus during consensus meeting

| Items suggested for re-evaluation | Preliminary consensus (eDelphi) | Support for re-evaluation | Re-evaluation* | Arguments in favour of item | Arguments against item | Comment |
| --- | --- | --- | --- | --- | --- | --- |
| Burning | out | 84% | yes | 'Burning' is different from 'pain', rated high by patients, and a more like a long-term outcome. | 'Burning' is a form of 'pain'. Its translation to other languages is problematic. |  |
| Sensitive skin | out | 28% | no | Patients know what 'sensitive skin' means and find it essential. | Does not reflect treatment effectiveness, unclear term. |  |
| Number of flares in a given time | in | 55% | yes | It is easy to detect flares in the clinic. A patient pointed out that they are important. | 'Flare' is a vague term. Flares can only be assessed during a consultation. Covered by signs and symptoms. Only 60% of eDelphi patients found flares essential. | Even though the threshold (80%) for re-evaluation was not reached, the discussion and consensus vote continued. Finally, only 63% voted against the eDelphi consensus. |
| Treatment tolerability | in | 58% | no | This item may show how much a patient likes the treatment or reflect compliance. | Does not reflect treatment effectiveness, rather related to safety and therefore outside the defined scope. | It was later questioned whether the 80% threshold was suitable for this group of decisions, but it was too late to revise them. |
| Cure | in | no vote | yes | Cure is achieved when the hands are free of eczema, for example by avoiding allergens and irritants. | Many types of chronic HE cannot be cured. Moreover, hands that are free of HE (due to avoiding triggers) is not the same as cure. Freedom from HE is covered by 'HE control'. | There was not enough time in LG0 to review this item and no vote concerning its evaluation. Finally, 87% voted against the eDelphi consensus. |
| Chronicity | in | no vote | yes | - | Covered by signs and symptoms, does not reflect treatment effectiveness. | There was not enough time in LG0 to review this item and no vote concerning its evaluation. Finally, 86.5% voted against the eDelphi consensus. |

* Re-evaluation means that the item was considered controversial and therefore underwent discussion and consensus vote during meeting (see Table 3 for results of the consensus vote).
